# A CO_2_-Responsive Imidazole-Functionalized Fluorescent Material Mediates Cancer Chemotherapy

**DOI:** 10.3390/pharmaceutics15020354

**Published:** 2023-01-20

**Authors:** Vo Thuy Thien Ngan, Po-Yen Chiou, Fasih Bintang Ilhami, Enyew Alemayehu Bayle, Yeong-Tarng Shieh, Wei-Tsung Chuang, Jem-Kun Chen, Juin-Yih Lai, Chih-Chia Cheng

**Affiliations:** 1 Graduate Institute of Applied Science and Technology, National Taiwan University of Science and Technology, Taipei 10607, Taiwan; 2 Department of Chemical and Materials Engineering, National University of Kaohsiung, Kaohsiung 81148, Taiwan; 3 National Synchrotron Radiation Research Center, Hsinchu 30076, Taiwan; 4 Department of Materials Science and Engineering, National Taiwan University of Science and Technology, Taipei 10607, Taiwan; 5 Advanced Membrane Materials Research Center, National Taiwan University of Science and Technology, Taipei 10607, Taiwan; 6 R & D Center for Membrane Technology, Chung Yuan Christian University, Chungli, Taoyuan 32023, Taiwan; 7 Department of Chemical Engineering and Materials Science, Yuan Ze University, Chungli, Taoyuan 32003, Taiwan

**Keywords:** anticancer agent, CO_2_ responsiveness, hydrophobic–hydrophilic transition, imidazole-containing rhodamine 6G, potent cytotoxicity

## Abstract

We present a breakthrough in the synthesis and development of functional gas-responsive materials as highly potent anticancer agents suitable for applications in cancer treatment. Herein, we successfully synthesised a stimuli-responsive multifunctional material (I-R6G) consisting of a carbon dioxide (CO_2_)-sensitive imidazole moiety and spirolactam-containing conjugated rhodamine 6G (R6G) molecule. The resulting I-R6G is highly hydrophobic and non- or weakly fluorescent. Simple CO_2_ bubbling treatment induces hydrophobic I-R6G to completely dissolve in water and subsequently form self-assembled nanoparticles, which exhibit unique optical absorption and fluorescence behaviours in water and extremely low haemolytic ability against sheep red blood cells. Reversibility testing indicated that I-R6G undergoes reversible CO_2_/nitrogen (N_2_)-dependent stimulation in water, as its structural and physical properties can be reversibly and stably switched by alternating cycles of CO_2_ and N_2_ bubbling. Importantly, in vitro cellular assays clearly demonstrated that the CO_2_-protonated imidazole moiety promotes rapid internalisation of CO_2_-treated I-R6G into cancer cells, which subsequently induces massive levels of necrotic cell death. In contrast, CO_2_-treated I-R6G was not internalised and did not affect the viability of normal cells. Therefore, this newly created system may provide an innovative and efficient route to remarkably improve the selectivity, safety and efficacy of cancer treatment.

## 1. Introduction

Chemotherapy drugs—either alone or in combination with other medicines or treatments—inhibit the ability of cancer cells to replicate [1]. However, chemotherapy is a systematic treatment and has a number of drawbacks, including non-specificity, that can lead to serious side effects and drug resistance over time [2]. Targeted therapy based on nanomedicines and controlled-release drug delivery systems have been widely studied as strategies to reduce the harmful side effects of conventional chemotherapy [3,4]. Targeted therapy approaches aim to employ therapeutics that respond to specific exogenous or endogenous conditions in cancer cells that are not present in healthy tissues [5]. By taking advantage of the differences between the inter- and intracellular environments of normal and cancer cells, the cytotoxic activity of nanomedicines or drug-loaded nanocarriers can be turned on or off by specific stimuli to induce tumour cell death [6]. Thus, the targeted cytotoxic activity of chemotherapeutic drugs can be controlled and regulated by specific stimuli in the external microenvironment of cancer cells, such as pH [7], temperature [8,9] and reactive oxygen species (ROS) [10,11], to potentially achieve desirable treatment outcomes. In addition to the previously mentioned stimuli, cancer cells also have significantly higher intracellular carbon dioxide (CO_2_) levels than normal cells, thus the microenvironment of cancer cells is hypercapnic [12,13]. CO_2_ is a vital physiological gas in cellular environments, and its concentrations dramatically affect physiological homeostasis and the pH of cells [13,14]. Excessive CO_2_ production is one of the main reasons for acidification of tumour tissues; the levels of CO_2_ are much higher in cancer cells (59–84 mmHg) than normal cells (35–37 mmHg) [15,16]. Helmlinger et al. described the relationship between the levels of CO_2_ and pH of solid tumours, and stated that production of CO_2_ may play a significant role in the acidification of the tumour environment due to penetration of CO_2_ into the cellular matrix on reaching a state of carbonic acid equilibrium [17]. Inspired by the influence of CO_2_ on cancer cells, we confidently propose that hypercapnia could be used as a stimulus for CO_2_-responsive nanomedicines that respond to the hypercapnic tumour microenvironment to subsequently induce potent toxicity in cancer cells and thereby improve the therapeutic efficacy of cancer treatment. Thus, exploration and development of this proposal may pave the road towards targeted and responsive nanomedicines that improve the effectiveness of cancer therapeutics and potentially generate a new class of biomedicines based on gas-sensitive materials.

The recent emergence of gas-sensing responsiveness has opened up new opportunities for the development of intelligent gas-responsive materials in different fields, including gas separation, drug encapsulation and drug release [18,19,20]. CO_2_-switchable stimuli-responsive materials have received the most attention, as CO_2_ is abundant, non-toxic and environmentally harmless, and the structural and material properties of these materials can be altered by simple CO_2_ bubbling of an aqueous solution [18]. When the environmental temperature of the solution is increased or the solution is bubbled with an inert gas such as nitrogen (N_2_), CO_2_-protonated materials return back to their original structure and state, which enables stable and reversible switching phenomena of their physical characteristics [21,22]. CO_2_-responsive materials typically contain amine-functional groups, such as tertiary amines, amidine, guanidine or imidazole, that have a tendency to react with CO_2_ to form charged ammonium bicarbonate in an aqueous environment [23,24,25]. For example, Yu et al. successfully achieved a series of imidazole-containing polyphenylsulfone membranes for gas separation that showed high selectivity towards CO_2_ due to the strong interaction between CO_2_ and the imidazole group [26]. Heldebrant et al. synthesised and developed a series of guanidine-containing compounds that can efficiently capture and release CO_2_ over multiple cycles without losing activity or selectivity [27]. A recent study in our laboratory demonstrated that the structural properties and fluorescence behaviour of water-soluble polythiophene with hydrophilic tertiary amine side-chains can be rapidly and reversibly tuned by CO_2_ bubbling, which results in significantly enhanced biocompatibility and fluorescence stability for in vitro and in vivo bioimaging [28]. Based on the examples above, the introduction of CO_2_-responsive molecular groups into functional materials could represent an efficient route to effectively modulate their physical characteristics and thus potentially create a variety of novel gas-responsive materials for a range of potential applications [18,19,20].

Rhodamine 6G (R6G), a water-soluble cationic dye capable of penetrating living cells and inhibiting cell proliferation, has long been studied and used in chemotherapy [29,30]. R6G is also widely used in bioimaging and as a probe due to its impressively high photostability and fluorescence quantum yield [31,32]. Warner et al. reported a series of functionalised R6G molecules that demonstrated potent selective anticancer activity against breast cancer cell lines, which were realised by alternating the anion moieties of the precursor R6G [33,34]. In addition, these modified R6G derivatives were taken up into cancer cells via endocytosis and subsequently induced cancer cell death, without causing significant adverse effects in normal cells [35,36]. Thus, R6G may be modified to confer multiple biofunctional properties and potentially generate effective anti-cancer drugs with potent cytotoxicity, and the fluorescent distribution of these drugs can be tracked in living cells. However, despite this potential, several challenges have limited the development of R6G for chemotherapy, such as its tendency to form large aggregates in aqueous media [37], lack of structural stability in biological environments and insufficient selective internalisation by cancer cells [38,39]. As a potential strategy to combat these multifaceted challenges, we reasonably speculated that the combination of R6G with a CO_2_-responsive imidazole group may potentially allow the development of intelligent anticancer drug systems with improved structural stability and potent anticancer cytotoxicity, and may also represent a strategy to enhance the overall efficacy and safety profile of medicines used for chemotherapy.

Our recent studies proved that the introduction of adenine moieties remarkably altered the amphiphilicity and fluorescence behaviour of R6G and promoted the co-assembly of uracil-functionalised supramolecular polymers into stable spherical nanogels. The resulting enhanced structural stability and selective delivery of the adenine-functionalised R6G drug into cancer cells eventually promoted rapid intracellular drug release and massive apoptotic cell death [38,39]. Based on these previous findings, we further extended our efforts to develop CO_2_-responsive functional anticancer drugs (I-R6G) by introducing imidazole groups into R6G, with the objectives of endowing CO_2_ responsiveness to R6G in an aqueous environment and also manipulating its structural stability and physical characteristics. The most important objective of this work was to explore the cytotoxic activity of I-R6G before and after CO_2_ treatment against normal and cancer cells and to assess the potential of CO_2_-treated I-R6G to enhance the efficacy of cancer treatment.

**Scheme 1:**
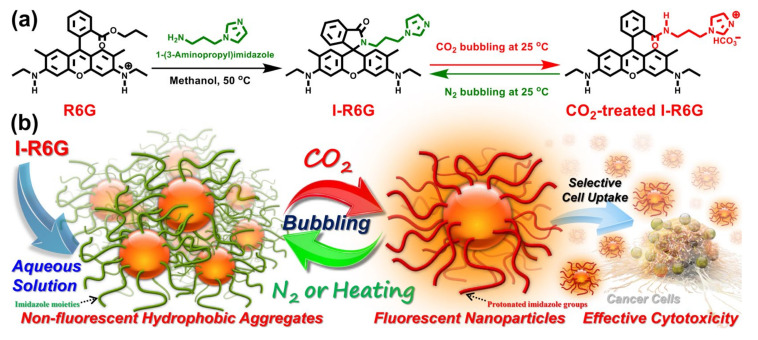
(**a**) Synthesis of CO_2_-responsive I-R6G from commercial R6G dye. (**b**) Illustration of the self-assembly and fluorescence switching behaviour of I-R6G in response to CO_2_ and N_2_ bubbling and the cancer cell-specific chemotherapeutic effects of CO_2_-treated I-R6G.

In this study, we successfully designed and synthesised CO_2_-responsive imidazole-functionalised I-R6G via a simple, one-step amidation reaction. The resulting I-R6G is highly hydrophobic and non-fluorescent in neutral and weakly acidic aqueous solutions (Scheme 1a). Due to the presence of the CO_2_-responsive imidazole moiety, I-R6G in water switches its solubility from hydrophobic to hydrophilic on CO_2_ bubbling and subsequently self-assembles into stable nanoparticles in water. These nanoparticles exhibit unique optical absorption and fluorescence properties and extremely low haemolytic activity against red blood cells. Furthermore, the structural features, surface charge, optical absorption and fluorescence characteristics of the I-R6G solution can be reversibly switched by repeated cycles of CO_2_ and N_2_ bubbling. More importantly, a series of in vitro assays demonstrated that CO_2_-treated I-R6G not only undergoes a high degree of selective cellular uptake in cancer cells, but also rapidly induces massive necrotic cell death in cancer cells without harming normal cells (Scheme 1b). As far as we are aware, this is the first report of a CO_2_-respective anticancer drug based on a combination of imidazole and R6G that exhibits highly selective cellular uptake and potent chemotherapeutic efficacy against cancer cells. Therefore, this newly developed system could potentially be used to develop a promising multifunctional anticancer drug to enhance the overall selectivity, efficacy and safety of a wide range of cancer drug therapies.

## 2. Materials and Methods

### 2.1. Chemicals and Materials

1-(3-Aminopropyl)imidazole (>98%) was purchased from Acros Organics (Geel, Belgium). Rhodamine 6G (R6G, dye content 99%), deuterated chloroform (CDCl_3_) and deuterium oxide (D_2_O) were obtained from Sigma-Aldrich Chemical (St. Louis, MO, USA) at the highest purity available. All organic solvents were of analytical grade and were purchased from TEDIA (Fairfield, OH, USA).

### 2.2. Reagents and Materials for In Vitro Cell-Based Assays

Phosphate-buffered saline (PBS), Dulbecco’s modified Eagle’s medium (DMEM), foetal bovine serum (FBS), penicillin-streptomycin, trypsin-EDTA, trypan blue, 3-(4,5-dimethylthiazol-2-yl)-2,5-diphenyl tetrazolium bromide (MTT), 4′,6-diamidino-2-phenylindole (DAPI), the Dead Cell Apoptosis Kit with Brilliant Violet-421™ Annexin V (BV421 Annexin V), and Ghost Dye™ Red 780 (DGR-780) were purchased from Thermo Fisher Scientific (Waltham, MA, USA). Sheep red blood cells (SRBCs) for the haemolysis assay were purchased from Cosmo Bio (Tokyo, Japan). Murine non-tumour NIH/3T3 fibroblasts, human HeLa cervical cancer cells and MG-63 osteosarcoma cancer cells were obtained from the American Type Culture Collection (Rockville, MD, USA) and cultured according to the supplier’s protocols.

### 2.3. Synthesis of Imidazole-Functionalised R6G (I-R6G)

1-(3-Aminopropyl)imidazole (1 mL, 8.38 mmol) and R6G (1 g, 2.09 mmol) were dissolved in 200 mL of methanol and refluxed at 50 °C for 72 h. Methanol was removed by rotary evaporation and the crude product was dissolved in diethyl ether (200 mL), followed by removal of insoluble impurities by vacuum suction filtration using a Büchner funnel. After removing diethyl ether by rotary evaporation and washing three times with deionised water, the obtained product was recrystallised in methanol/water mixtures and dried overnight in a vacuum oven at 30 °C. A pink crystal powder was obtained at a product yield of 72% (0.78 g).

### 2.4. Preparation of CO_2_- and N_2_-Bubbled Aqueous Solution of I-R6G

I-R6G in water was directly bubbled with CO_2_ or N_2_ gases. Briefly, aqueous solutions of I-R6G in vials were directly bubbled with CO_2_ or N_2_ at constant flow rates (50 cc/s). The solutions obtained were used directly for various measurements or characterisations.

### 2.5. Characterisations

*Fourier transform infrared (FTIR) and proton/carbon nuclear magnetic resonance (^1^H- and ^13^C-NMR) spectra:* We obtained FTIR spectra using a PerkinElmer Spectrum Two IR spectrometer (Buckinghamshire, UK) over the scan range between 600–4000 cm^−1^ at a resolution of 2.0 cm^−1^. To further elucidate the chemical structure of I-R6G, ^1^H- and ^13^C-NMR spectra were recorded using a Bruker AVIII instrument (Billerica, MA, USA) at 500 MHz in deuterated solvents.

*Mass spectrometry:* The actual molecular mass of I-R6G was analysed in methanol using both high- and low-resolution electrospray ionisation mass spectrometry (HR- and LR-MS; VG Platform, Fisons Instruments, Altrincham, UK). The mass data and spectra were recorded in both positive and negative ion mode.

*Elemental analysis (EA):* A Flash 2000 Elemental Analyzer (Thermo Fisher Scientific, Voltaweg, The Netherlands) was used to carry out CHN elemental analyses. Simultaneous determination of the elements C, H, and N was based on full combustion of the samples at up to 1200 °C in an oxygen atmosphere.

*Ultraviolet-visible (UV-Vis) and photoluminescence (PL) spectra:* The UV-Vis optical absorption and fluorescence spectra of I-R6G before and after CO_2_ or N_2_ bubbling were determined using a PL spectrometer (Hitachi F4500, Tokyo, Japan) and a Jasco V-730 UV-Vis spectrophotometer (Hachioji, Tokyo, Japan) at 25 °C.

*Dynamic light scattering (DLS) and zeta potentials:* The hydrodynamic diameter, size distribution, surface charge and polydispersity index (PDI) of aqueous I-R6G solutions (0.1 mg/mL) before and after CO_2_ bubbling were assessed using a Nano Brook 90Plus PALS instrument (Brookhaven, Holtsville, NY, USA) equipped with a 632 nm He-Ne laser beam at a fixed scattering angle of 90°. All samples were incubated at 25 °C for at least 30 min before DLS measurements.

*Atomic force microscopy (AFM) and scanning electron microscopy (SEM):* Thin films of CO_2_-treated I-R6G solution were prepared on silicon wafers using a spin coater and vacuum dried at 30 °C for 24 h. The surface morphology of the thin films was assessed using a tapping-mode AFM (NX10; AFM Park Systems, Suwon, Republic of Korea) equipped with a standard commercial probe made of silicon (125 nm). The microstructure of the thin films was further determined using a field-emission SEM (JSM-6500F, JEOL, Tokyo, Japan).

*Single-crystal X-ray diffractometry:* A crystal powder of I-R6G was obtained by the anti-solvent crystallisation method. Briefly, 3 mg of I-R6G was dissolved in methanol, and deionised water was added until the transparent solution turned opaque. The solution was heated at 50 °C until the solution turned transparent, stored in a 4 °C refrigerator overnight, and the crystals were collected by vacuum suction filtration and dried in a vacuum dryer overnight. Finally, the geometry of the I-R6G crystals was determined using a D8 Venture single-crystal X-ray diffraction system equipped with Cu and Mo InCoatec microfocus X-ray sources (Bruker, Karlsruhe, Germany).

### 2.6. Cell Culture Conditions

NIH/3T3, HeLa and MG-63 cells were cultured in T-75 culture flasks containing DMEM supplemented with 10% FBS and 1% penicillin-streptomycin in a 37 °C incubator in a humidified 5% CO_2_ atmosphere.

### 2.7. Haemolysis Assays

The haemolytic activity of R6G, I-R6G and CO_2_-treated I-R6G were assayed using SRBCs. Briefly, 1 mL of SRBCs and 0.5 mL of PBS were mixed in a microcentrifuge, centrifuged at 12,000 rpm for 15 min, and the plasma supernatant was extracted. The pellet was vortexed and centrifuged in 1.5 mL of PBS three times until the supernatant was clear. Then, various quantities of R6G, I-R6G or CO_2_-treated I-R6G (1, 2.5, 5, 10, 25 and 50 µg/mL) were added to the SRBC solutions (500 µL). PBS and Triton X-100 solution (1%) were used as negative and positive controls, respectively. All samples were placed in a 5% CO_2_ incubator at 37 °C for 4 h, centrifuged, before 100 mL of the supernatants were transferred to a 96-well plate, and the absorbance values were quantified using an ELISA reader (BioTek, Winooski, VT, USA) at 540 nm. The haemolysis index was calculated using the equation:

$$\mathrm{Haemolysis}\left(\%\right)=\frac{A_{\mathrm{sample}}-A_{\mathrm{negative}}}{A_{\mathrm{positive}}-A_{\mathrm{negative}}}\times 100$$

where A represents the optical density (OD) of the test sample, positive control (1% Triton X-100) or negative control (PBS).

### 2.8. In Vitro Cytotoxicity Assays

NIH/3T3, HeLa and MG-63 cells were seeded into 96-well plates at 1 × 10^6^ cells per well in 100 μL DMEM culture media for 24 h, then incubated with pristine or CO_2_-treated I-R6G, and various concentrations of curcumin (0.01 to 100 μg/mL) for 24 h at 37 °C. Then, 20 μL of MTT solution (5 mg/mL) in PBS was added to each well and incubated for 4 h. The media containing unreacted dye was then carefully removed, the blue formazan crystals were dissolved in 100 μL dimethyl sulfoxide solution, and the absorbance values were determined using a microplate reader (ELx800; BioTek, Winooski, VT, USA) set at 570 nm. For the MTT assay, the cells without any treatment were used as the control group to assess cytotoxic effects of pristine and CO_2_-treated I-R6G.

### 2.9. Cellular Internalisation of Pristine and CO_2_-Treated I-R6G

HeLa and NIH/3T3 cells were seeded into glass dishes at an initial density of 2 × 10^5^ cells/well in 2 mL DMEM culture media, incubated for 24 h, washed thrice with PBS, and then the original media was replaced with fresh DMEM media (pH 7.4) containing pristine or CO_2_-treated I-R6G. The cells were cultured for 3, 12 or 24 h, washed thrice with PBS, fixed in 4% paraformaldehyde for 30 min, stained using blue-fluorescent DAPI for 15 min to visualise nuclei, washed thrice with PBS and examined by confocal laser scanning microscopy (CLSM; iRiS Digital Cell Imaging System, Logos Biosystems, Republic of Korea).

### 2.10. Assessment of the Cellular Uptake of Pristine and CO_2_-Treated I-R6G by Flow Cytometry

Approximately 2 × 10^5^ HeLa or NIH/3T3 cells were seeded into 6-well plates in DMEM media (2 mL), incubated overnight, then incubated with pristine or CO_2_-bubbled culture media containing I-R6G at 37 °C for 1, 6, 12 or 24 h. The cells were rinsed twice with PBS, detached with 0.25% trypsin-EDTA (0.5 mL) and harvested by centrifugation at 1500 rpm for 3 min. The cell pellet was washed with PBS, centrifuged and re-suspended in ice cold PBS (0.5 mL), and the cells were examined by flow cytometry (FACSAria^TM^ III; BD Biosciences, San Jose, CA, USA). Flow cytometry data were analysed using FlowJo software (FlowJo, LLC, Ashland, OR, USA, https://www.flowjo.com/).

### 2.11. Detection and Quantitative Analysis of Programmed Cell Death Induced by Pristine and CO_2_-Treated I-R6G

Approximately 2 × 10^5^ HeLa or NIH/3T3 cells in DMEM (2 mL) were seeded into 6-well plates, incubated overnight, and treated with pristine or CO_2_-bubbled culture media containing I-R6G (2 mL) for 1, 6, 12 or 24 h. Then, the cells were washed with PBS, detached using 0.25% trypsin-EDTA, centrifuged at 1500 rpm for 3 min, the supernatant was washed with PBS, centrifuged, and the cells were re-suspended in binding buffer (100 μL) in flow cytometry tubes. The cells were stained using the BV421 Annexin V and GDR-780 detection Kit (Thermo Fisher Scientific, Waltham, MA, USA). Briefly, GDR-780 (1 μL) was added, incubated in the dark at ambient temperature for a minimum of 15 min, BV421 Annexin V (5 μL) was added and incubated under the same conditions for 30 min, and then binding buffer (400 μL) was added and the cells were analysed by flow cytometry (FACSAria^TM^ III; BD Biosciences). Untreated cultured cells were employed as controls.

### 2.12. Statistical Analysis

All experiments were performed in triplicate and repeated at least three times; the mean ± standard error (±SD) values are reported.

## 3. Results and Discussion

**Figure 1:**
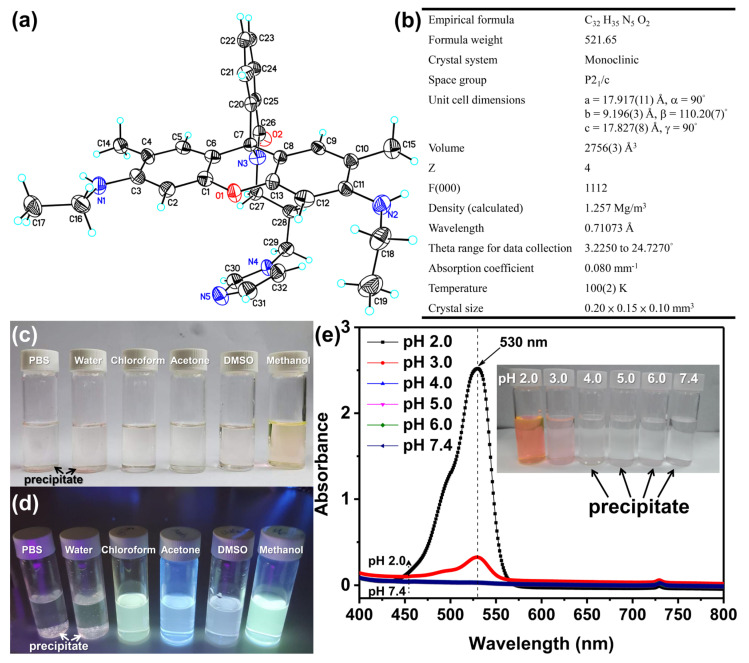
(**a**) Single crystal structure of I-R6G determined using single-crystal X-ray diffraction. (**b**) Crystallographic data and structure refinement details for the crystal structure of I-R6G. Photographs of I-R6G in various solvents at 25 °C under (**c**) natural light and (**d**) long-wavelength UV lamp illumination. (**e**) UV-Vis spectra of I-R6G in aqueous solutions with pH values of 2.0–7.4 at 25 °C. The inset photograph shows the solubility of I-R6G in aqueous solutions with different pH values.

The primary aim of this study was to develop CO_2_-responsive anticancer nanomedicines to improve the selectivity, effectiveness and safety of chemotherapy, as depicted in Scheme 1. Imidazole-functionalised R6G (I-R6G) was obtained through a simple, one-step amidation reaction of R6G with excess 1-(3-aminopropyl)imidazole under mild heating without a catalyst, resulting in a crystalline pink powder with an acceptable yield of 72% (Scheme 1a). During the reaction, an intermediate form with an amide bond undergoes intramolecular self-cyclisation to create a spirolactam functional group in the structure of I-R6G, in which an amide group bonds to the C9 atom of the xanthene conjugate group of I-R6G by nucleophilic attack [40,41,42], leading to the formation of a bulky heterocyclic aromatic structure. The recrystallised I-R6G exhibited the expected structural features (including molecular structure, weight and elemental composition), as confirmed by Fourier transform infrared (FTIR), proton/carbon nuclear magnetic resonance (^1^H- and ^13^C-NMR), mass spectrometry (MS) and elemental analysis (EA) (see Supporting Information for more detail, Figures S1–S4 and Table S1). In addition, single-crystal X-ray diffraction clearly revealed that the crystal structure of I-R6G exists as a locked spirolactam moiety (Figure 1a). The spirolactam moiety is positioned almost orthogonally to the plane of the xanthene moiety in I-R6G, indicating a stable spatial arrangement between the spirolactam and xanthene moieties. Details of the crystal data and structure refinement for I-R6G are summarised in Figure 1b and the Supplementary Crystallographic Information File. After confirming the chemical structure of I-R6G, the solubility of I-R6G in different aqueous solutions and organic solvents was evaluated in order to explore the effect of introducing the imidazole and spirolactam moieties on the solubility of R6G. As shown in Figure 1c,d, I-R6G was highly soluble in common organic solvents and also displayed unique fluorescence under a long wavelength ultraviolet (UV) lamp. However, I-R6G was very difficult to dissolve in water and phosphate-buffered saline (PBS), even after the solution was heated to 60 °C for 1 day, suggesting that the presence of the imidazole and spirolactam moieties in the molecular structure of I-R6G profoundly affect its solubility and amphiphilic properties compared to water-soluble pristine R6G. Similar trends in the solubility of I-R6G were also observed in ultraviolet-visible (UV-Vis) and photoluminescence (PL) spectra. As presented in Figure S5, I-R6G in organic solvents exhibited clear absorption and florescence peaks, but did not exhibit any characteristic UV-Vis and PL peaks in water or PBS, further confirming that I-R6G is hydrophobic and is highly soluble in a wide range of polar and non-polar organic solvents, i.e., R6G reacted with 1-(3-aminopropyl)imidazole was fully converted from hydrophilic to hydrophobic. Therefore, this intriguing finding motivated our inquisitiveness to explore the environmental-responsive behaviour of I-R6G in aqueous solution.

Imidazole molecules tend to form a hydrophilic imidazole salt upon protonation of the tertiary amine in the imidazole ring under aqueous acidic conditions [43]. Likewise, the non- or weakly fluorescent closed spirolactam ring in R6G derivatives can be opened to produce highly fluorescent ring-opened amide forms under acidic conditions, which confers highly sensitive “turn-on” fluorescent sensing behaviour towards acid environments [44,45]. Therefore, we reasonably speculated that acidic aqueous conditions may significantly alter the water solubility and fluorescence properties of I-R6G. In order to confirm our hypothesis, I-R6G was dissolved in aqueous solutions with pH values ranging from 2.0 to 7.4; the pH was adjusted by adding small amounts of dilute hydrochloric acid (HCl). Surprisingly, after stirring at 25 °C for 1 day, I-R6G was completely insoluble in aqueous solutions over the pH range from 4.0 to 7.4 (upper right inset in Figure 1e), suggesting I-R6G is strongly acid-resistant, possibly due to the presence of robustly stable crystal structures. The solution gradually changed from light pink to bright orange as the pH was further decreased to 3.0 and 2.0, indicating aqueous solutions with pH values lower than 3.0 prompt a rapid reaction between I-R6G and hydrochloric acid to form hydrophilic I-R6G containing a hydrochloride imidazole salt and ring-opened amide moiety (Scheme S1). As a result of low pH altering the solubility of I-R6G, UV-Vis and PL spectra revealed that the maximum intensity of the I-R6G absorption peak at 530 nm significantly increased as the pH was decreased from 3.0 to 2.0 (Figure 1e), while the characteristic fluorescence peak markedly red-shifted from 555 nm to 568 nm and substantially increased in intensity from 137 to 2508 a.u. (Figure S6). Thus, the increased water solubility and fluorescence properties of I-R6G could be attributed to protonation of the imidazole moiety and structural transformation of the spirolactam form of I-R6G to an amide form under highly acidic conditions [46,47]. Thus, the combination of imidazole and spirolactam moieties synergistically improves the water solubility and fluorescence behaviour of I-R6G under aqueous acidic conditions. Based on these results, we reasonably concluded that I-R6G does not significantly undergo a weak acid-induced solubility transition and fluorescence switch owing to its highly hydrophobic nature. While the pH-sensitive imidazole and spirolactam moieties enhance the pH-responsive capacity of I-R6G, HCl barely diffuses into the interior of the I-R6G structure, and thus I-R6G maintains it structural integrity and stability in mildly acidic aqueous solution.

**Figure 2:**
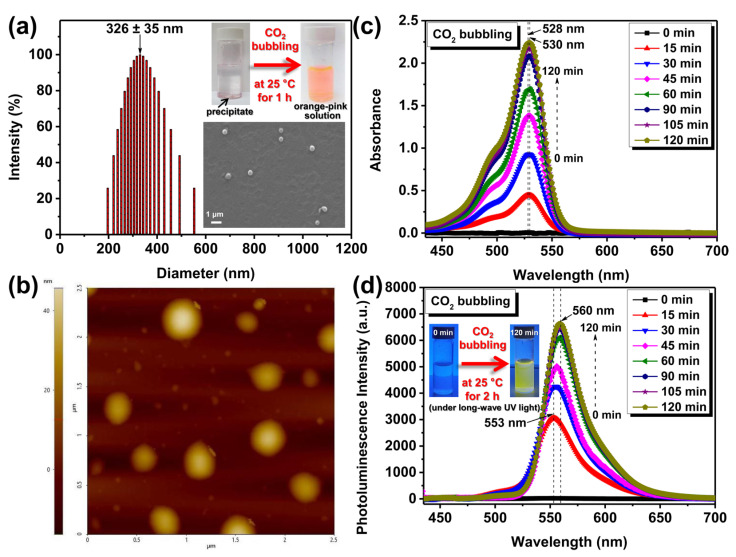
(**a**) DLS size distribution curve of I-R6G in water after CO_2_ bubbling at 25 °C. The upper right inset photographs show the effect of CO_2_ bubbling on the solubility of I-R6G in water. The bottom right inset is a SEM image of spin-coated CO_2_-treated I-R6G nanoparticles obtained at 25 °C. (**b**) AFM image of spin-coated CO_2_-treated I-R6G nanoparticles obtained at 25 °C. (**c**) UV-Vis and (**d**) PL spectra of I-R6G (0.1 mg/mL) in water obtained at 25 °C over time after various periods of CO_2_ bubbling. The upper left inset photographs in (**d**) show the fluorescence behaviour of aqueous I-R6G solution under long-wavelength UV lamp illumination before and after CO_2_ bubbling.

Water-insoluble I-R6G did not exhibit pH-induced structural changes or fluorescence responses in a weakly acidic aqueous environment. However, recent studies have reported that imidazole and imidazole-functionalised materials exhibited high selectivity and excellent adsorption capacity for CO_2_ in both aqueous solution and solid state [48,49,50]. Thus, we speculated that bubbling CO_2_ into I-R6G in water may promote a rapid reaction between the imidazole moiety of I-R6G and CO_2_ to form a charged ammonium bicarbonate group, and subsequently improve the water solubility and fluorescent properties of I-R6G [28,51]. Thus, we performed digital photography and dynamic light scattering (DLS) measurements at 25 °C to investigate the effects of CO_2_ treatment on the solubility and self-assembly behaviour of I-R6G in water. Hydrophobic I-R6G (0.1 g) was a colourless aqueous solution with pink I-R6G precipitate before CO_2_ bubbling. As expected, the 1 mL solution completely changed to a homogeneous orange-pink solution after bubbling CO_2_ for 60 min at 25 °C (see the photographs in the upper right corner inset of Figure 2a). These results imply that I-R6G was completely protonated and converted into a hydrophilic molecule by CO_2_ bubbling, i.e., the formation of a charged ammonium bicarbonate group in the imidazole moiety promoted the dissolution of I-R6G in water. In addition, as shown in Figure 2a, DLS analysis further revealed that I-R6G in water bubbled with CO_2_ for 60 min had a mean hydrodynamic diameter of 326 ± 35 nm, mean zeta potential of 17.22 ± 1.01 mV and pH of 4.75 ± 0.06, suggesting that formation of the hydrophilic protonated imidazole moiety increased the water solubility of I-R6G and its ability to form self-assembled nano-objects. In order to validate these results, scanning electron microscopy (SEM) and atomic force microscopy (AFM) were used to explore the morphological and microstructural features of spin-coated I-R6G thin films before and after CO_2_ bubbling. As indicated in Figure 2a (lower right corner) and 2b, the SEM and AFM images confirmed that CO_2_-bubbled I-R6G was composed of uniform spherical structures with a smooth surface ranging in diameter from 150 nm to 300 nm; these values are slightly smaller than the hydrodynamic diameter obtained by DLS due to expansion of the particles by the thin electric dipole layer on their surface [52]. In contrast, pristine I-R6G exhibited large crystal aggregates with an uneven surface and wide size distribution ranging from approximately 2 µm to over 10 µm (Figure S7). These observations indicated that the formation of self-assembled nano-objects by I-R6G after CO_2_ bubbling in water was possibly due to strong amphiphilic repulsion between the hydrophobic π-conjugated aromatic ring and hydrophobic protonated imidazole moiety inducing efficient structured packing into water-soluble nanoparticles [28]. The SEM and AFM results are consistent with the DLS data, and further confirm the formation of charged ammonium bicarbonate groups in the imidazole moiety of I-R6G after CO_2_ bubbling significantly alters the structural features and self-assembly ability of I-R6G and enables the formation of spherical nanoparticles in water.

We also observed that CO_2_ bubbling significantly changed the pH of the I-R6G solution to 4.75 ± 0.06, which may induce the ring-opening reaction within I-R6G spirolactam and lead to remarkable turn-on fluorescence (Scheme S1) [44,45]. Thus, UV-Vis and PL were employed to further investigate the effects of the duration of CO_2_ bubbling on the absorption and fluorescence signals of I-R6G in water at 25 °C. As presented in Figure 2c,d, after only 15 min CO_2_ bubbling, the characteristic absorbance and fluorescence peaks of 0.1 mg/mL I-R6G in water were observed at 528 nm and 553 nm, respectively. When the CO_2_ bubbling was extended to 120 min, these peaks gradually red-shifted from 528 nm to 530 nm and 553 nm to 560 nm, and their absorbance and fluorescence intensities also gradually increased from 0.4 to 2.3 and 3175 to 6731, respectively. This indicates that CO_2_ and I-R6G gradually react to form protonated imidazole groups in water as the duration of CO_2_ bubbling increases, and the resulting intermolecular interactions between the protonated imidazole groups and water molecules eventually improve the water solubility of I-R6G [53,54]. Moreover, the pH decreased gradually to around 4.7 when CO_2_ was bubbled through I-R6G solution for 120 min, and the structure of I-R6G changed from a non- or weakly fluorescent ring-closed spirolactam form into a highly fluorescent ring-opened amide form under acid conditions, resulting in a dramatic enhancement of fluorescence (see the photographs in the upper left inset in Figure 2d) [44,45]. Further insight into the effects of CO_2_ bubbling on the solubility of I-R6G in water before and after CO_2_ bubbling were obtained using ^1^H-NMR spectroscopy. As illustrated in Figure S8, ^1^H-NMR (0.1 mg of I-R6G in 1 mL of D_2_O) indicated that CO_2_-bubbled I-R6G showed clear characteristic proton peaks whereas no detectable peaks were observed for I-R6G before CO_2_ bubbling, further confirming that hydrophobic I-R6G can be converted to a hydrophilic state via CO_2_ bubbling. Overall, these experiments confirmed our hypothesis that the structural transformation, self-assembly and fluorescence behaviour of I-R6G can be effectively controlled by CO_2_ bubbling to tailor the amphiphilic and photophysical properties of I-R6G. Thus, these intriguing results further piqued our curiosity to explore the reversibility and stability of the changes in CO_2_-treated I-R6G in response to various external environmental stimuli in water.

**Figure 3:**
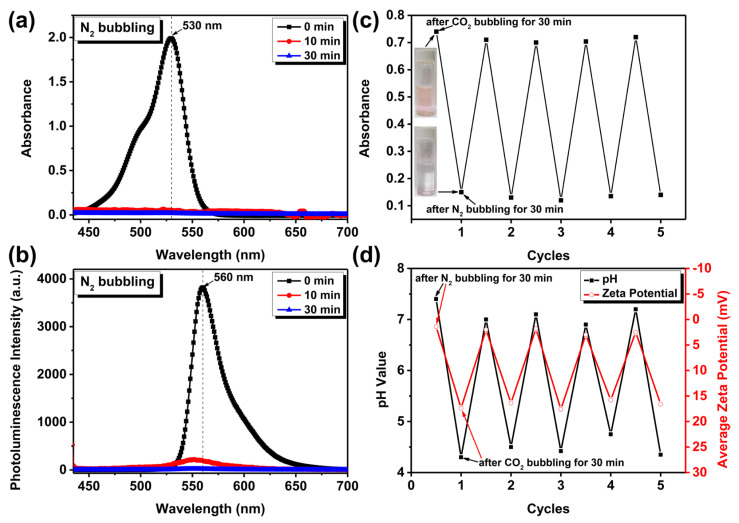
(**a**) UV-Vis and (**b**) PL spectra of CO_2_-treated I-R6G (0.1 mg/mL) in water bubbled with N_2_ for various periods of time at 25 °C. Reversible changes in the (**c**) absorption intensity and (**d**) pH and zeta-potential values of aqueous I-R6G solution (0.1 mg/mL) over five alternating cycles of CO_2_/N_2_ bubbling at 25 °C. Each cycle lasted 1 h, with 30 min CO_2_ bubbling and 30 min N_2_ bubbling. The left inset photographs in (**c**) show the effects of CO_2_ and N_2_ bubbling on the solubility of I-R6G in water.

The production of charged ammonium bicarbonate groups within I-R6G molecules in water by CO_2_ bubbling is a transient reaction that can be reversed by adding an inert gas or changing the temperature [24]. The most common gas used to remove CO_2_ from solution is N_2_, which makes up approximately 78% of air [25]. Therefore, deprotonation of CO_2_-treated I-R6G by N_2_ bubbling was evaluated by UV-Vis and PL spectroscopy at 25 °C. As shown in Figure 3a, I-R6G solution bubbled with CO_2_ for 1 h was subjected to N_2_ bubbling at 25 °C. Interestingly, after only 10 min of N_2_ bubbling, the maximum absorbance wavelength of the UV-Vis spectra for I-R6G at 530 nm completely returned to the original state of hydrophobic I-R6G, with no characteristic peaks. The absorbance signal remained absent for I-R6G when N_2_ bubbling was extended to 30 min, confirming the ability of a short period of N_2_ bubbling to eliminate CO_2_ from I-R6G. The PL spectra in Figure 3b exhibited similar trends, as the fluorescence intensity substantially decreased after bubbling N_2_ for only 10 min and entirely disappeared after 30 min of N_2_ bubbling, further indicating that the structure of CO_2_-protonated I-R6G after N_2_ bubbling rapidly transforms from a ring-opened amide to a spirolactam form, resulting in the disappearance of the fluorescence signal [44,45] and a change from a hydrophilic to hydrophobic structure.

Temperature may also affect the structural stability of protonated I-R6G in water and restore its original structure. Hence, after bubbling CO_2_ for 60 min, protonated I-R6G was immediately evaluated by PL spectroscopy at various temperatures (4, 25 and 45 °C) over time. The maximum fluorescence intensity of I-R6G at 555 nm exhibited a small reduction from 3740 to 2870 after 24 h of monitoring at 4 °C, whereas the maximum fluorescence peaks slightly blue-shifted and their intensities considerably reduced at 25 °C and 45 °C (Figure S9). For instance, the fluorescence peak of I-R6G gradually blue-shifted from 560 nm to 556 nm and reduced in intensity by more than half after 24 h at 25 °C, while the fluorescence peak completely disappeared after 24 h at 45 °C. These results demonstrate that increasing the temperature of the CO_2_-treated I-R6G solution accelerates the removal of CO_2_ from I-R6G and the return of the structure to its original state, i.e., production of the hydrophobic and low-fluorescence spirolactam ring. Thus, CO_2_-treated I-R6G in water has a notably higher CO_2_ entrapment stability at low temperature (4 °C) than at room-temperature (25 °C) or elevated temperature (45 °C) and when treated with N_2_, further demonstrating that the rate of removal of CO_2_ from the structure of I-R6G is linearly proportional to the temperature of the environment and can be increased by N_2_ bubbling.

Next, to explore the stability of the gas-triggered switchable amphiphilicity and explore the potential reuse of I-R6G as an efficient gas-absorbing material for reversible CO_2_ capture and release, we further evaluated the reversible hydrophilic and hydrophobic characteristics of I-R6G in water upon alternating CO_2_/N_2_ bubbling by UV-Vis spectroscopy at 25 °C. As shown in Figure 3c, the changes in the absorption intensity after five cycles of bubbling with CO_2_ and N_2_ for 30 min each at 25 °C indicated that I-R6G in water undergoes stable CO_2_/N_2_-dependent stimulation and the absorption intensity can reversibly and stably switch from around 0.7 to 0.1 after each cycle of CO_2_/N_2_ treatment. Thus, alternating CO_2_/N_2_ bubbling of aqueous I-R6G solution led to highly stable structural transformations between a hydrophobic spirolactam form and a hydrophilic ring-opened amide form, as reflected by macroscopic observation of alternating formation of the precipitate and orange–pink solution (see the photographs in the left inset in Figure 3c and video in Supplementary Movie S1, respectively). Similarly, as shown in Figure 3d, the pH and zeta potential values of the I-R6G solution underwent stable and reversible switching between approximately 17 mV and near-neutral zeta potential and pH 4.4 and neutral pH upon alternating CO_2_/N_2_ bubbling, again demonstrating that gas-responsive I-R6G undergoes a highly stable structural transition and hydrophobic–hydrophilic switching behaviour in water, even though pristine I-R6G is completely insoluble in water. Furthermore, when the concentration of I-R6G in water was decreased from 0.1 mg/mL to 0.01 mg/mL, the reversible switching phenomenon on alternating CO_2_/N_2_ bubbling was also clearly observed in the UV-Vis absorption spectra (Figure S10). These results imply that low concentrations of I-R6G in water exhibit concentration-independent CO_2_/N_2_-responsive ability, with reproducible protonation/deprotonation of the imidazole moiety accompanied by “dissolution–precipitation” cycling behaviour upon alternate cycles of CO_2_ and N_2_ bubbling [55]. To date, no reports have described a CO_2_-sensitive R6G system that exhibits highly stable and reversible structural and physical characteristics in water upon alternating cycles of CO_2_ and N_2_ bubbling. Thus, our findings that CO_2_-protonated I-R6G possesses unique amphiphilic behaviour and fluorescence performance in water encouraged us to study the biocompatibility of I-R6G before and after CO_2_ bubbling.

**Figure 4:**
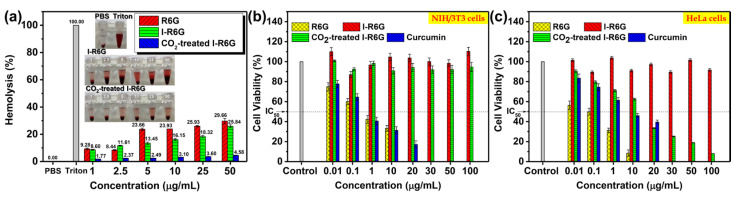
(**a**) In vitro haemolytic SRBC assays. The inset photographs show SRBCs after incubation with varying concentrations (1–50 μg/mL) of R6G and pristine or CO_2_-treated I-R6G for 4 h. In vitro cell viability assays of (**b**) NIH/3T3 and (**c**) HeLa cells after incubation with varying concentrations (0.01–100 μg/mL) of R6G and pristine or CO_2_-treated I-R6G for 24 h.

Biomedical materials must be highly biocompatible with the normal biological environment to ensure their suitability for biomedical applications [56]. Therefore, we explored the biocompatibility of I-R6G with blood before and after CO_2_ bubbling using the sheep red blood cell (SRBC) haemolysis assay. SRBCs were utilised to induce structural destabilisation of self-assembled I-R6G and lead to dissolution of the I-R6G in media [57]. As shown in Figure 4a and the attached photographs, R6G and I-R6G solutions at concentrations ranging from 1 μg/mL to 50 μg/mL exhibited strong haemolytic activity towards SRBCs (approximately 27% and 26% haemolysis at 50 µg/mL, respectively). In contrast, the CO_2_-treated I-R6G solution exhibited dramatically lower haemolytic activity; even concentrations as high as 50 μg/mL showed low haemolytic activity of 4.6%. Thus, CO_2_-treated I-R6G appears to exhibit extremely low haemolytic activity and high biocompatibility with blood, thus this material holds potential for in vivo biomedical applications [58,59]. The presence of the protonated imidazole (or charged ammonium bicarbonate) group within the structure probably enhances the structural stability of I-R6G and endows low haemolytic activity [60].

After confirming the haemolytic activity of CO_2_-protonated I-R6G, the cytotoxic activity of I-R6G before and after CO_2_ bubbling towards normal NIH/3T3 fibroblasts, HeLa cervical cancer cells and MG-63 osteosarcoma cells was explored using the colorimetric 3-(4,5-dimethylthiazol-2-yl)-2,5-diphenyl tetrazolium bromide (MTT) assay. As shown in Figure 4b,c and Figure S11, after 24 h of culture, pristine I-R6G at concentrations ranging from 0.01 μg/mL to 100 μg/mL had no significant cytotoxic effects on the normal or cancer cells. However, the hydrophilic precursor R6G exhibited extremely high cytotoxicity towards NIH/3T3 and HeLa cells, with half-maximal inhibitory concentrations (IC_50_) of 0.59 ± 0.22 and 0.10 ± 0.09 µg/mL, respectively. These results indicate that the hydrophobic nature of I-R6G almost completely inhibits its cytotoxic activity against both cell lines. In order to verify the non-cytotoxicity of I-R6G, commercial curcumin with a negative zeta potential (around −26 mV) was used as a model hydrophobic anticancer drug to evaluate cytotoxic activity [61,62]. Curcumin exerted potent cytotoxic effects against NIH/3T3 and HeLa cells, with IC_50_ values of 0.61 ± 0.34 μg/mL and 7.02 ± 1.86 μg/mL, respectively. These results are in complete contrast with the effects of hydrophobic I-R6G, and further indicate that the non-cytotoxic effects of I-R6G can be attributed to both its hydrophobic nature and near-neutral zeta potential (or surface charge), which significantly inhibit the cellular uptake and limit the cytotoxicity of I-R6G compared to hydrophobic curcumin. Surprisingly, when I-R6G bubbled with CO_2_ for 60 min was incubated with the cells for 24 h, over 95% of normal NIH/3T3 fibroblasts remained viable. However, the viability of HeLa and MG-63 cancer cells gradually reduced with the concentration of CO_2_-treated I-R6G, with notable IC_50_ values of 15 ± 2.02 μg/mL in HeLa cells and 9 ± 1.06 μg/mL in MG-63 cells (Figure 4b,c and Figure S11). These results reveal that CO_2_-treated I-R6G has a high affinity and subsequently leads to rapid cytotoxic death in cancer cells [63,64]. Thus, formation of the protonated imidazole moiety within CO_2_-bubbled I-R6G may critically enhance its selective cellular internalisation and ability to promote cell death in cancer cells, but remarkably reduces the harmful effects of I-R6G in normal cells. These differences may possibly be due to differences in the surface charge of normal cells and cancer cells [65]. Therefore, CO_2_-responsive I-R6G may function as a highly efficient anticancer drug to dramatically improve the selectivity, safety and efficacy of chemotherapy.

**Figure 5:**
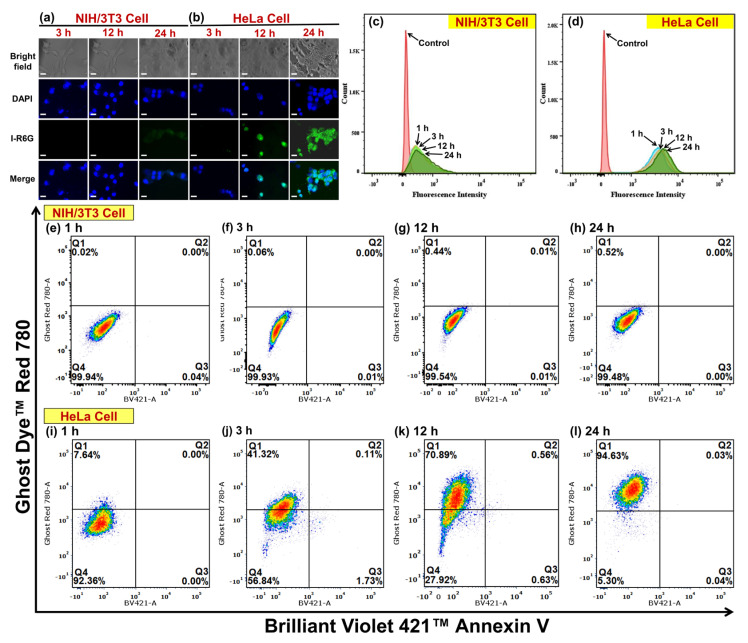
CLSM images of (**a**) NIH/3T3 and (**b**) HeLa cells cultured with CO_2_-treated I-R6G at 37 °C for 3, 12 or 24 h. The scale bar in each image represents 20 μm. Flow cytometry histogram profiles of (**c**) NIH/3T3 and (**d**) HeLa cells cultured with CO_2_-treated I-R6G at 37 °C for 1, 3, 12 or 24 h. Representative flow cytometry dot plot diagrams of (**e**–**h**) NIH/3T3 and (**i**–**l**) HeLa cells incubated with CO_2_-treated I-R6G at 37 °C for 1, 3, 12 or 24 h, then double-stained with BV421 Annexin V and GDR-780.

The intriguing potent cytotoxic action of CO_2_-protonated I-R6G in cancer cells piqued our curiosity to directly evaluate cellular internalisation of I-R6G before and after CO_2_ bubbling in NIH/3T3 and HeLa cells by confocal laser scanning microscopy (CLSM). 4′,6-Diamidino-2-phenylindole (DAPI) is a nuclear staining reagent that emits bright blue fluorescence; I-R6G displays green fluorescence. The characteristic green fluorescence of pristine I-R6G could not be observed in NIH/3T3 and HeLa cells after 24 h culture (Figure S12). Surprisingly, HeLa cells cultured with CO_2_-treated I-R6G exhibited strong green fluorescence in the cytoplasm after 12 h, and this fluorescence signal was significantly brighter and progressively moved toward the nucleus after 24 h culture. In contrast, only very low green-fluorescence intensity was observed in NIH/3T3 cells after 24 h culture with CO_2_-treated I-R6G (Figure 5a,b). These observations are consistent with the MTT assay and confirmed that CO_2_-treated I-R6G is selectively and progressively internalised by cancer cells, mainly due to a strong complementary electrostatic interaction between positively charged CO_2_-treated I-R6G and negatively charged HeLa cells [66], whereas the internalisation of I-R6G by normal NIH/3T3 cells is significantly limited. To further assess the cellular uptake of I-R6G before and after CO_2_ bubbling, we conducted flow cytometric measurements to quantitatively and qualitatively analyse the extent of internalisation of CO_2_-treated I-R6G by NIH/3T3 and cells. As shown in Figure 5c,d, after culture with CO_2_-treated I-R6G for 24 h, NIH/3T3 cells displayed no significant increase in fluorescence intensity, whereas the fluorescence intensity of I-R6G within HeLa cells progressively increased during the co-culture period. These data demonstrate that CO_2_-treated I-R6G exhibits highly selective affinity for HeLa cells, as specific electrostatic interactions between CO_2_-treated I-R6G and the surface of the HeLa cells [66] enhance intracellular accumulation of I-R6G, which subsequently induces progressive loss of cell viability and functionality. In contrast to CO_2_-treated I-R6G, flow cytometry analysis showed the fluorescence intensity of NIH/3T3 and HeLa cells treated with hydrophilic precursor R6G gradually enhanced as the incubation time increased from 1 h to 24 h (Figure S13), indicating that R6G is not selectively taken up by cancer cells. In addition, R6G exhibited a greater degree of more rapid cellular uptake by HeLa cells than NIH/3T3 cells, probably because of the differences in surface affinity between these cells and R6G [67].

Next, we quantitatively evaluated how the CO_2_-protonated imidazole moiety promotes selective cellular internalisation of I-R6G. As shown in Figure S14, the fluorescence intensity of CO_2_-treated I-R6G in NIH/3T3 cells only slightly increased from approximately 370 at 1 h to 770 at 24 h. In contrast, the fluorescence intensity of CO_2_-treated I-R6G in HeLa cells progressively increased from 3700 at 1 h to nearly 5100 after 24 h. Thus, the rate of uptake of CO_2_-treated I-R6G was approximately seven times higher in HeLa cells than NIH/3T3 cells, which is highly consistent with the MTT assay and CLSM analyses. Collectively, these results demonstrate that the self-assembly behaviour, structural charge and amphiphilic features of I-R6G can be efficiently manipulated by CO_2_ bubbling to promote rapid, selective uptake by HeLa cancer cells and only minimal uptake in NIH/3T3 normal cells. Furthermore, these results confirm that the CO_2_-protonated imidazole moiety plays an important role in conferring the selective internalisation, accumulation and strong cytotoxic activity of I-R6G in cancer cells. Thus, CO_2_-sensitive I-R6G could potentially improve the overall safety and efficacy of chemotherapy.

In order to determine the mechanisms by which CO_2_-treated I-R6G mediates potent cytotoxicity in HeLa cells, a double staining flow cytometric assay was employed to estimate the percentages of live, apoptotic and necrotic cells after incubation of NIH/3T3 or HeLa cells with pristine or CO_2_-treated I-R6G for different periods of time. As indicated in Figure 5e–l and Figure S15, double Ghost Dye™ Red 780 (GDR-780) and Brilliant Violet-421™ Annexin V (BV421 Annexin V) staining were employed to quantify necrotic cells (BV421 Annexin V negative and GDR-780 positive; upper left quadrant; Q1), late apoptotic cells (BV421 Annexin V positive and GDR-780 positive; upper right quadrant; Q2), early apoptotic cells (BV421 Annexin V positive and GDR-780 negative; lower right quadrant; Q3) and live cells (BV421 Annexin V negative and GDR-780 negative; lower left quadrant; Q4) [38,39,68,69]. Up to 99 and 98% of NIH/3T3 and HeLa cells remained viable after culture with pristine I-R6G for 24 h, respectively, proving once again that pristine I-R6G does not exert any cytotoxic effects in either cell line (Figure S15). Similarly, as shown in Figure 5e–h, over 99% of NIH/3T3 cells cultured with CO_2_-treated I-R6G were still alive after 24 h, clearly indicating that the CO_2_-protonated imidazole moiety in CO_2_-treated I-R6G does not induce cytotoxicity in normal cells. However, when HeLa cells were cultured with CO_2_-treated I-R6G, the proportions of necrotic cells progressively increased over time (Figure 5i–l). After 24 h of culture, around 95% of HeLa cells were necrotic, while almost no cells were apoptotic and only approximately 5% of cells were alive. These results clearly demonstrate that the CO_2_-protonated imidazole moiety effectively promotes selective uptake of the self-assembled I-R6G nanoparticles by cancer cells via passive diffusion, and the I-R6G subsequently induces massive levels of necrotic cell death [33,70,71]. Despite the high levels of necrosis, almost no early or late apoptotic cells were observed, further indicating that CO_2_-treated I-R6G can selectively penetrate the membrane of cancer cells and subsequently induces highly potent cytotoxic effects within the intracellular environment as CO_2_-treated I-R6G is gradually internalised into the nucleus. Therefore, even though I-R6G is highly hydrophobic, its structural, amphiphilic and fluorescence characteristics and self-assembly behaviour can be efficiently manipulated in an aqueous environment using CO_2_ bubbling. Thus this newly created gas-responsive functional material based on a combination of a CO_2_-sensitive imidazole group and spirolactam-containing R6G holds the potential to rapidly induce necrosis and remarkably enhance cancer treatment, while minimally harming normal cells.

## 4. Conclusions

We successfully established a highly efficient route to synthesise a CO_2_-responsive material that functions as a highly potent anticancer agent, exhibits environmental stimuli-responsive fluorescence and is well suited to a wide variety of biomedical applications, including biological sensing and cancer treatment. This new multifunctional anticancer drug (I-R6G) consists of a CO_2_-responsive imidazole moiety conjugated to spirolactam-containing R6G and was prepared using a simple, efficient one-step synthetic route. I-R6G exhibits extremely poor solubility and is non-fluorescent in water, even in weakly acidic aqueous solution (pH 4.0–7.4). Due to the presence of the CO_2_-responsive imidazole and pH-sensitive spirolactam moieties, hydrophobic I-R6G can completely dissolve in water after CO_2_ bubbling. The resulting self-assembled spherical nanostructures have an average diameter of approximately 300 nm, possess unique optical absorption and fluorescence characteristics and exhibit extremely low haemolytic activity towards SRBCs. Furthermore, I-R6G in water has the unique ability to undergo reversible and stable CO_2_/N_2_-dependent switching of its pH, zeta potential, absorption and fluorescence properties upon alternating cycles of CO_2_ and N_2_ bubbling, which efficiently manipulates the structural and physical properties of I-R6G. This combination of unique structural transformations is rarely found in traditional fluorescent organic materials. Thus, the reversible physical CO_2_/N_2_-responsive switching of the absorption/fluorescence properties and haemolytic activity of I-R6G suggest this material may enable the development of high-performance water-soluble fluorescent nanomaterials for biomedical imaging and sensing applications. In addition, in vitro cytotoxicity MTT assays indicated that CO_2_-treated I-R6G exhibits highly potent cytotoxicity towards cancer cells, without minimally harming healthy cells. Control R6G did not exhibit significantly different cytotoxicity towards normal or cancer cells, which demonstrates that the CO_2_-protonated imidazole moiety critically enhances the selective internalisation, accumulation and cytotoxic activity of I-R6G towards cancer cells. Importantly, CLSM and double staining flow cytometric assays of cellular internalisation and the mechanisms of cytotoxicity clearly confirmed that CO_2_-treated I-R6G can selectively penetrate into cancer cells and subsequently induces massive levels of necrotic cell death, but is not significantly internalised and thus does not induce cell death in normal cells. Therefore, this newly created system clearly illustrates that the combination of CO_2_-responsive imidazole and pH-sensitive spirolactam moieties within the structure of I-R6G provides a multifunctional stimuli-responsive material with high potential for biomedical sensing and imaging, and this system could also potentially be combined with a functional nanocarrier to significantly improve the selectivity, safety and efficacy of cancer chemotherapy.

## Data Availability

Not applicable.

## Supplementary Materials

The following are available online at https://www.mdpi.com/article/10.3390/pharmaceutics15020354/s1, Scheme S1: Chemical opening of the spirolactam ring of I-R6G to the ring-opened amide form under acidic conditions, Figure S1: FTIR spectra of R6G and I-R6G at 25 °C, Figure S2: ^1^H-NMR spectrum of I-R6G in deuterated chloroform (CDCl_3_) obtained at 25 °C, Figure S3: ^13^C-NMR spectrum of I-R6G in CDCl_3_ obtained at 25 °C, Figure S4: (a) Low- and (b) high-resolution mass spectra of I-R6G, Figure S5: (a) UV-Vis and (b) PL spectra of I-R6G in various solvents at 25 °C, Figure S6: PL spectra of aqueous I-R6G solution in solutions with various pH values at 25 °C, Figure S7: (a) SEM and (b) AFM images of spin-coated 1-R6G thin films obtained at 25 °C, Figure S8: ^1^H-NMR spectra of I-R6G (0.1 mg/mL) in deuterium oxide (D_2_O) before and after CO_2_ bubbling at 25 °C, Figure S9: PL spectra of 0.1 mg/mL CO_2_-bubbled I-R6G in water at (a) 4 °C, (b) 25 °C and (c) 45 °C over time, Figure S10: (a) UV-Vis spectra of I-R6G (0.01 mg/mL) in water after CO_2_ bubbling over time at 25 °C. (b) UV-Vis spectra of CO_2_-treated I-R6G (0.01 mg/mL) in water after N_2_ bubbling over time at 25 °C. (c) Reversible changes in the absorption intensity of aqueous I-R6G solution (0.01 mg/mL) upon five alternating cycles of CO_2_/N_2_ bubbling at 25 °C; each cycle lasted 1 h, with CO_2_ and N_2_ bubbling for 30 min each, Figure S11: Cell viability of MG-63 cells in vitro after incubation with varying concentrations of pristine or CO_2_-treated I-R6G (0.01–100 μg/mL) for 24 h, Figure S12: CLSM images of (a) NIH/3T3 and (b) HeLa cells cultured with pristine I-R6G at 37 °C for 3, 12 or 24 h. The scale bar in each image represents 20 μm, Figure S13: Flow cytometry histogram profiles of (a) NIH/3T3 and (b) HeLa cells cultured with R6G at 37 °C for 1, 3, 6 or 24 h, Figure S14: Flow cytometric analysis of the changes in the fluorescence intensities of NIH/3T3 and HeLa cells incubated with CO_2_-treated I-R6G at 37 °C for 1, 3, 6 or 24 h, Figure S15: Representative dot plot diagrams generated by flow-cytometric analysis of (a–d) NIH/3T3 and (e–h) HeLa cells incubated with pristine I-R6G at 37 °C 1, 3, 12 or 24 h, then double stained with BV421 Annexin V and GDR-780, Table S1: Elemental content of I-R6G. Movie S1: Alternating CO_2_/N_2_ bubbling of aqueous I-R6G solution.
