# Supplementary material for: A CO2-Responsive Imidazole-Functionalized Fluorescent Material Mediates Cancer Chemotherapy
Source: Pharmaceutics. 2023 Jan 20;15(2):354. doi: 10.3390/pharmaceutics15020354 (PMC9959563; doi:10.3390/pharmaceutics15020354)
Supplement: Supplementary file 1 [file pharmaceutics-15-00354-s001.zip › Supporting Information.pdf]

Supporting Information

A CO_2-Responsive Imidazole-Functionalized Fluorescent Material Mediates Cancer Chemotherapy

Vo Thuy Thien Ngan [1], Po-Yen Chiou [1], Fasih Bintang Ilhami [1], Enyew Alemayehu Bayle [1], Yeong-Tarng Shieh [2], Wei-Tsung Chuang [3], Jem-Kun Chen [4], Juin-Yih Lai [1,5,6,7] and Chih-Chia Cheng [1,5,*]

[1] Graduate Institute of Applied Science and Technology, National Taiwan University of Science and Technology, Taipei 10607, Taiwan. E-mail: cccheng@mail.ntust.edu.tw.

[2] Department of Chemical and Materials Engineering, National University of Kaohsiung, Kaohsiung 81148, Taiwan.

[3] National Synchrotron Radiation Research Center, Hsinchu 30076, Taiwan.

[4] Department of Materials Science and Engineering, National Taiwan University of Science and Technology, Taipei 10607, Taiwan.

[5] Advanced Membrane Materials Research Center, National Taiwan University of Science and Technology, Taipei 10607, Taiwan.

[6] R&D Center for Membrane Technology, Chung Yuan Christian University, Chungli, Taoyuan 32023, Taiwan.

[7] Department of Chemical Engineering and Materials Science, Yuan Ze University, Chungli, Taoyuan 32003, Taiwan.

* Corresponding Author

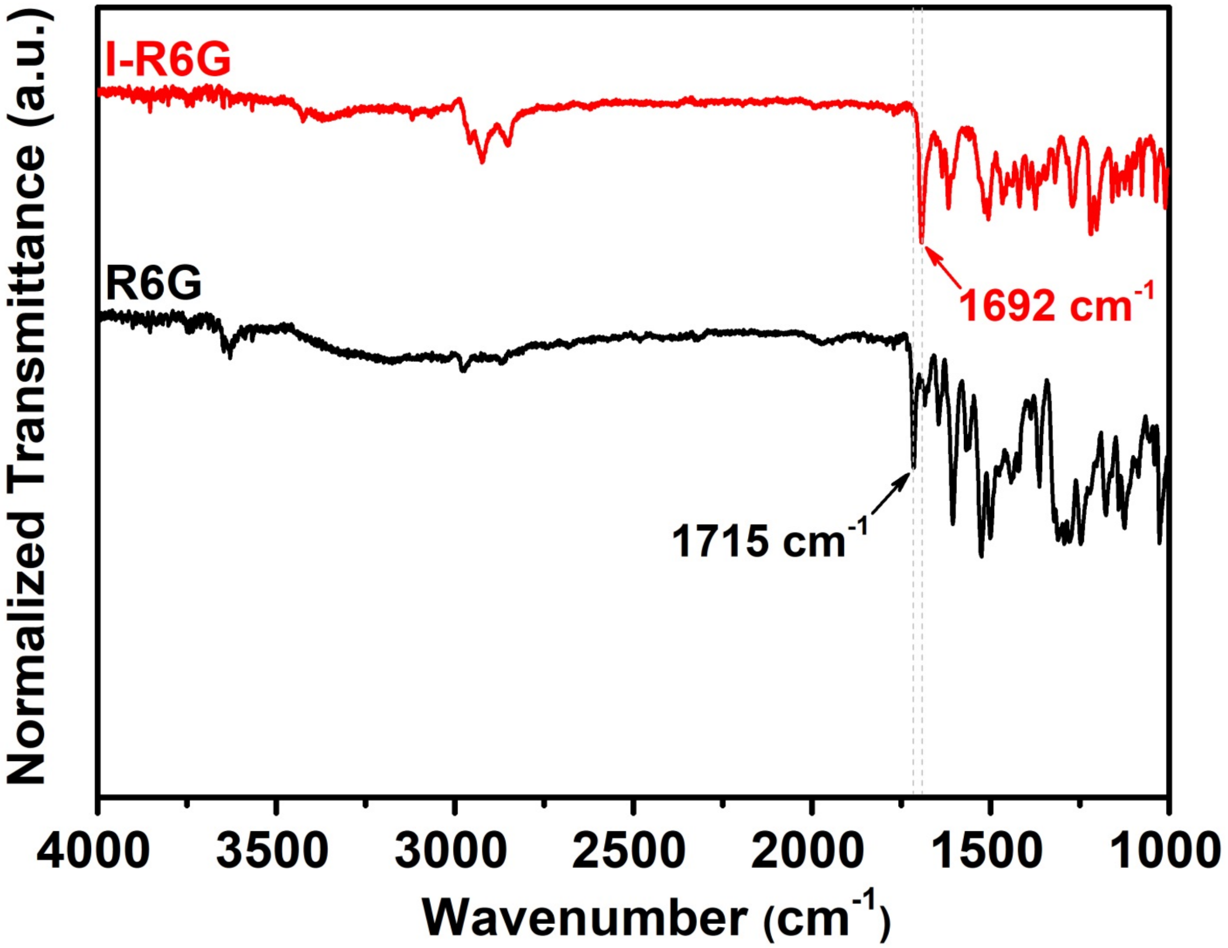

Figure S1: FTIR spectra of R6G and I-R6G at 25 °C.

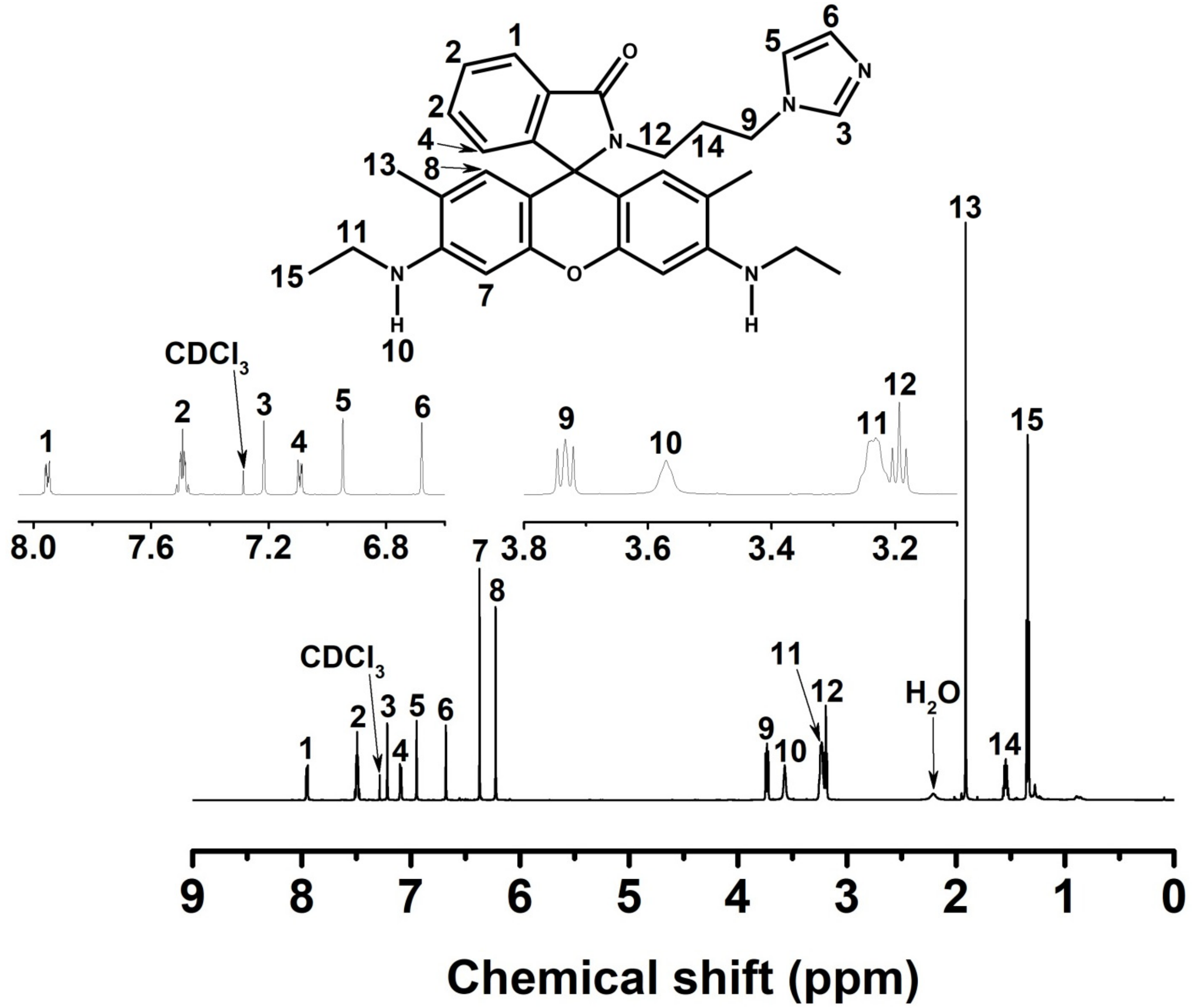

Figure S2: ^{1}H-NMR spectrum of I-R6G in deuterated chloroform ($CDCl_3$) obtained at 25 °C.

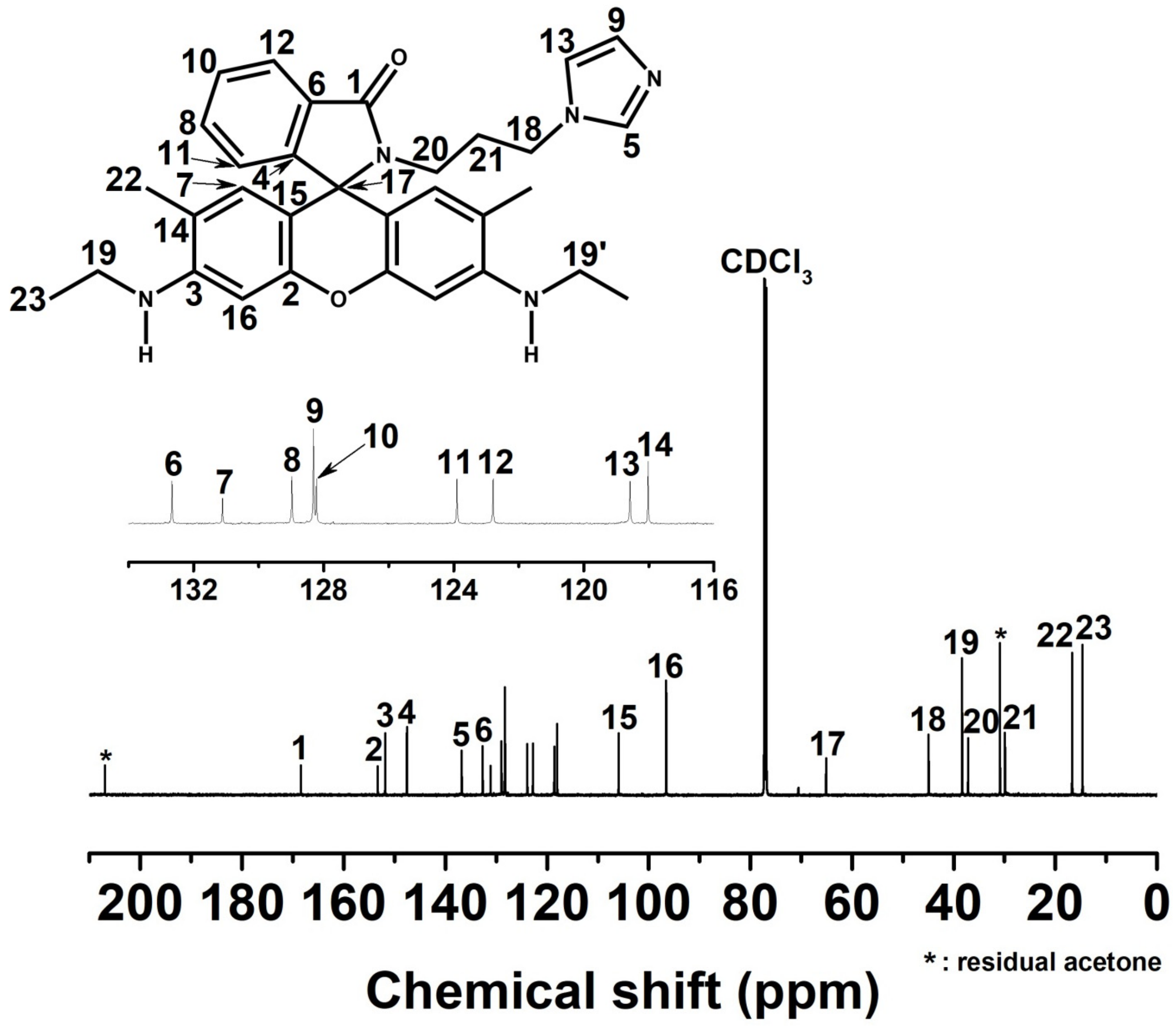

Figure S3: ^{13}C-NMR spectrum of I-R6G in $CDCl_3$ obtained at 25 °C.

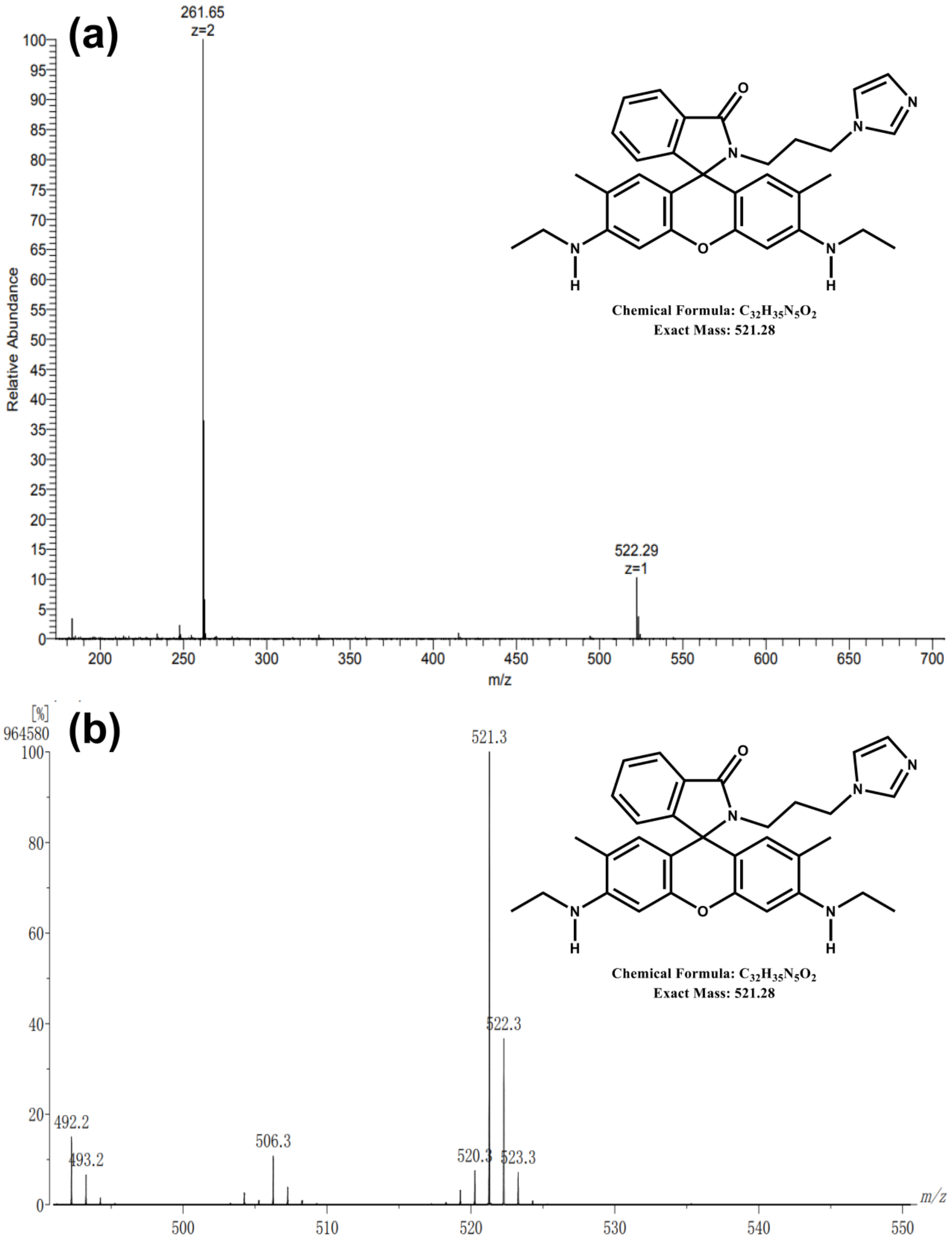

Figure S4: (a) Low- and **(b)** high-resolution mass spectra of I-R6G.

Table S1: Elemental content of I-R6G.

Compound		C (%)	H (%)	N (%)
I-R6G	Found	72.014	6.758	12.787
	Theoretical	72.08	6.95	12.97

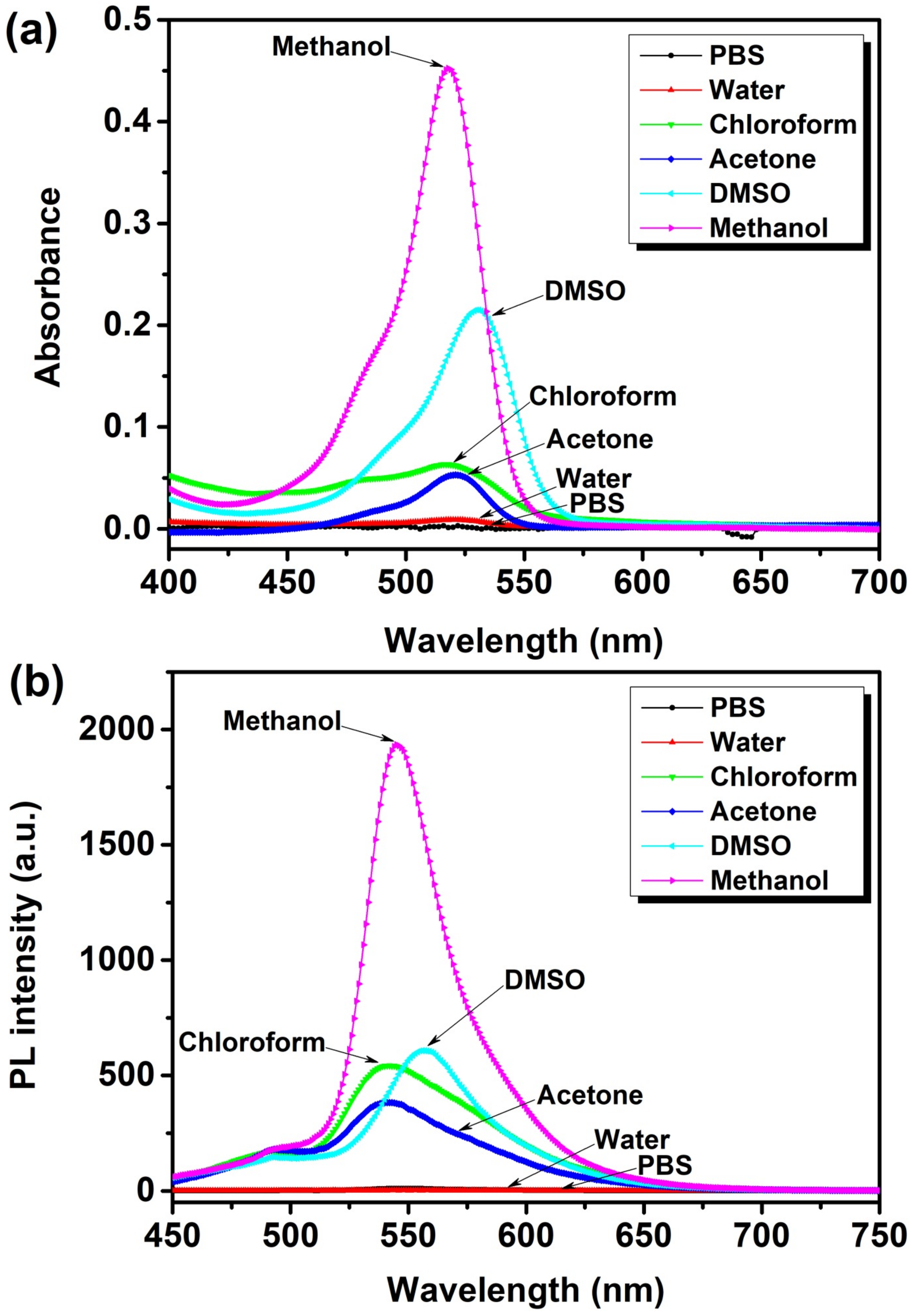

Figure S5: (a) UV-Vis and **(b)** PL spectra of I-R6G in various solvents at 25 °C.

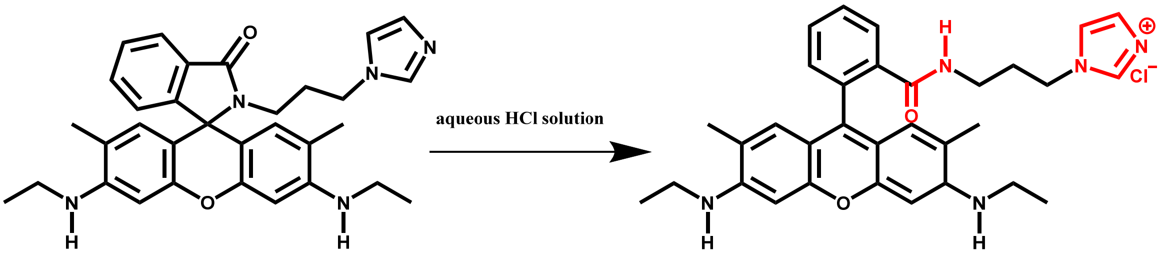

Scheme S1: Chemical opening of the spirolactam ring of I-R6G to the ring-opened amide form under acidic conditions.

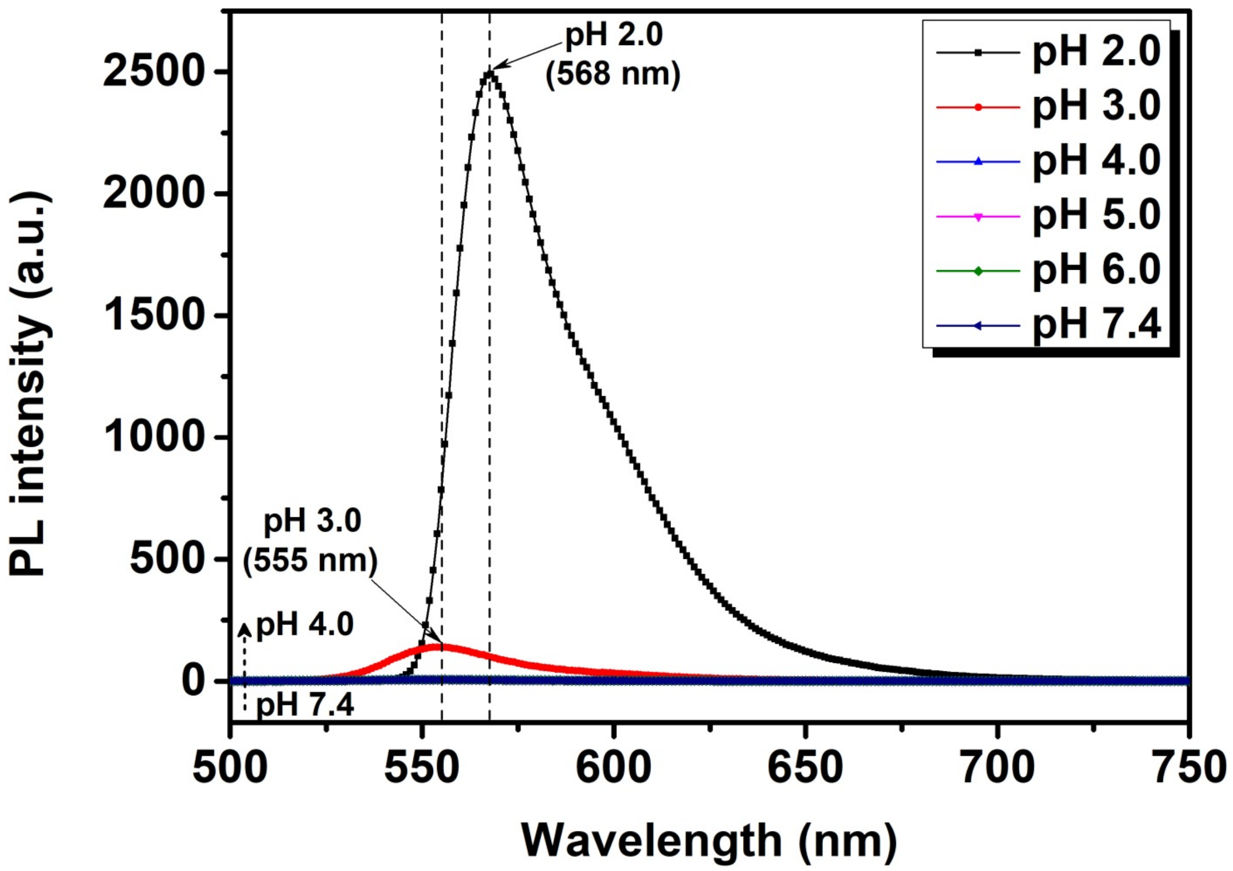

Figure S6: PL spectra of aqueous I-R6G solution in solutions with various pH values at 25 °C.

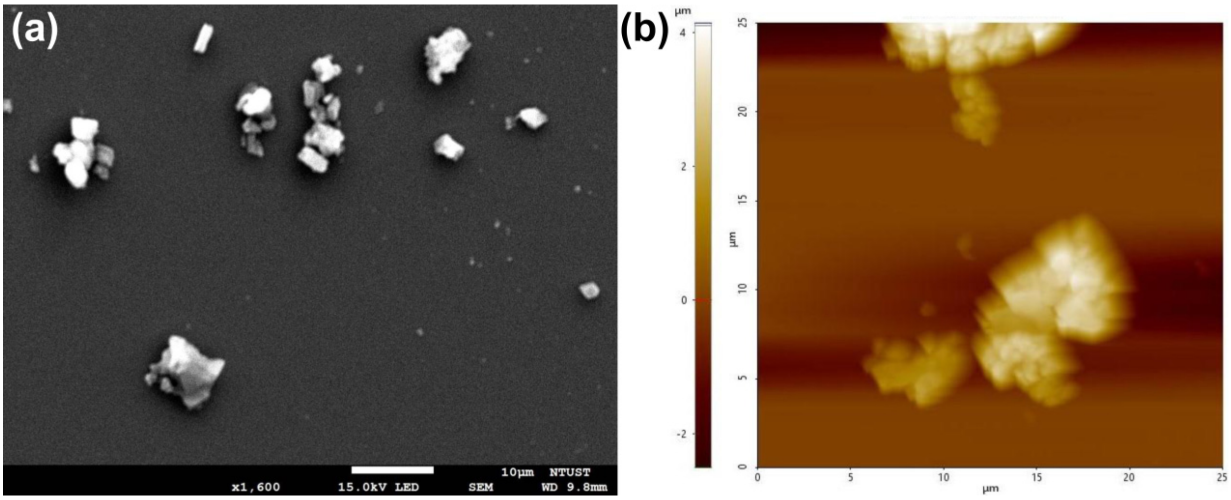

Figure S7: (a) SEM and **(b)** AFM images of spin-coated 1-R6G thin films obtained at 25 °C.

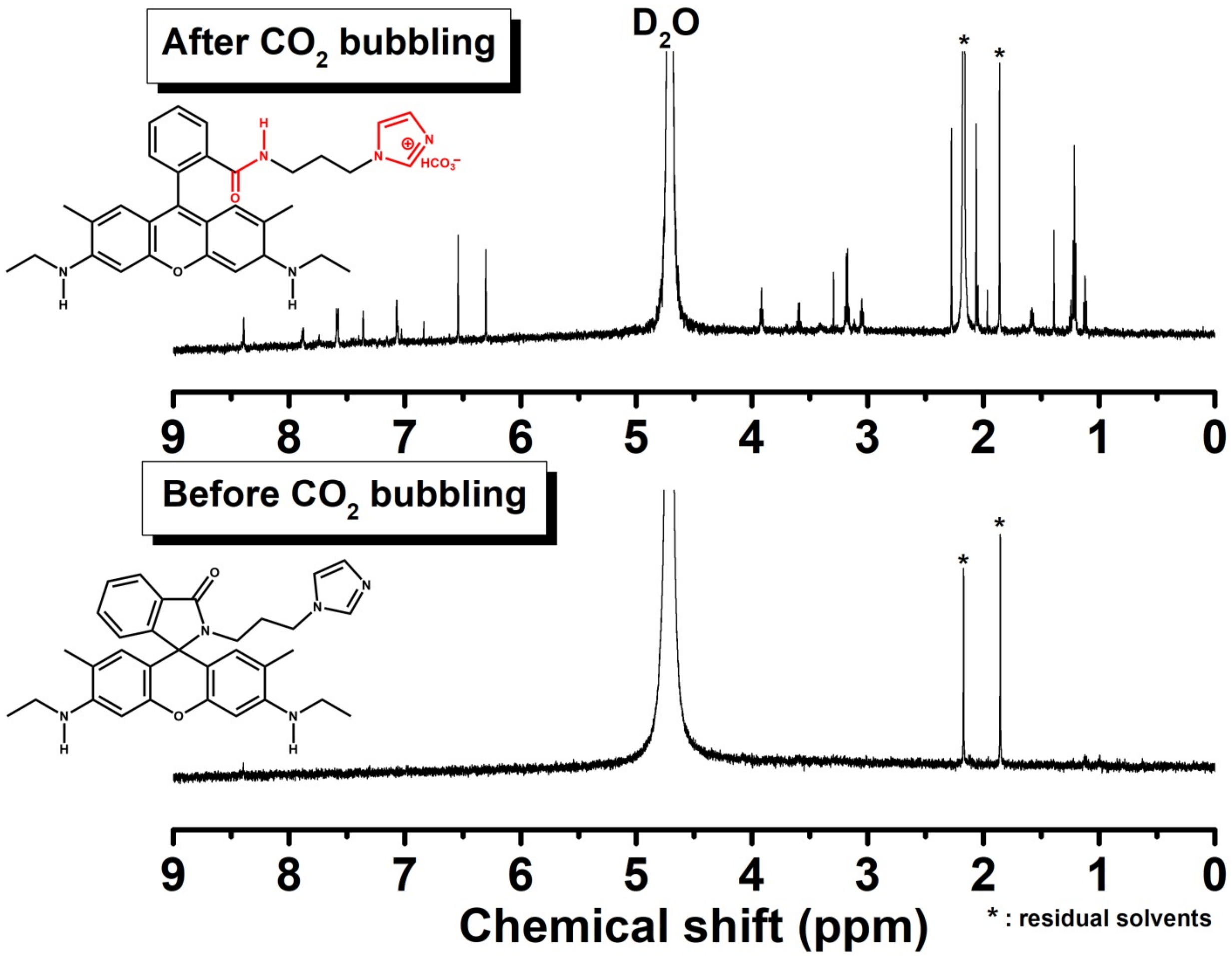

Figure S8: ^{1}H-NMR spectra of I-R6G (0.1 mg/mL) in deuterium oxide (D_2O) before and after CO_2 bubbling at 25 °C.

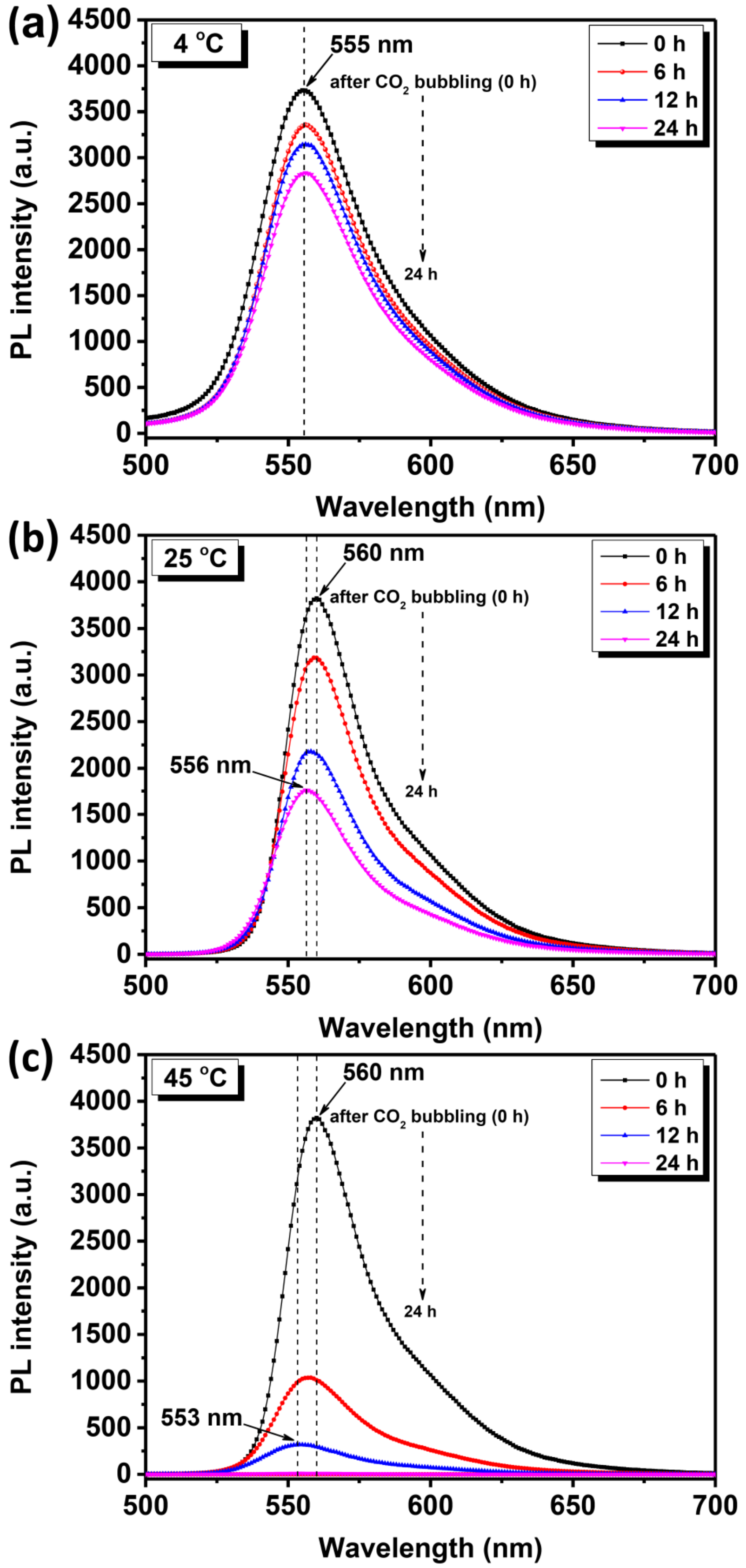

Figure S9: PL spectra of 0.1 mg/mL CO_2-bubbled I-R6G in water at **(a)** 4 °C, **(b)** 25 °C and **(c)** 45 °C over time.

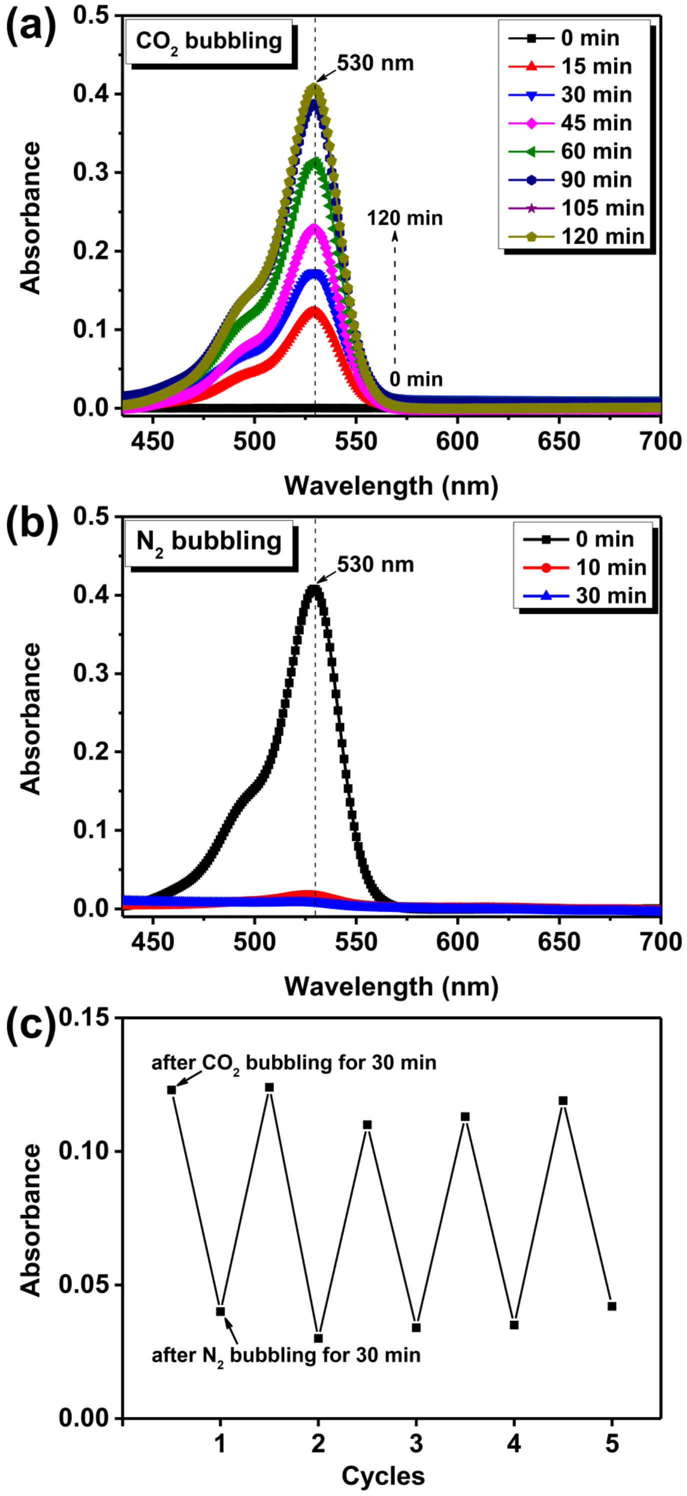

Figure S10: (a) UV-Vis spectra of I-R6G (0.01 mg/mL) in water after CO_2 bubbling over time at 25 °C. **(b)** UV-Vis spectra of CO_2-treated I-R6G (0.01 mg/mL) in water after N_2 bubbling over time at 25 °C. **(c)** Reversible changes in the absorption intensity of aqueous I-R6G solution (0.01 mg/mL) upon five alternating cycles of CO_2/N_2 bubbling at 25 °C; each cycle lasted 1 h, with CO_2 and N_2 bubbling for 30 min each.

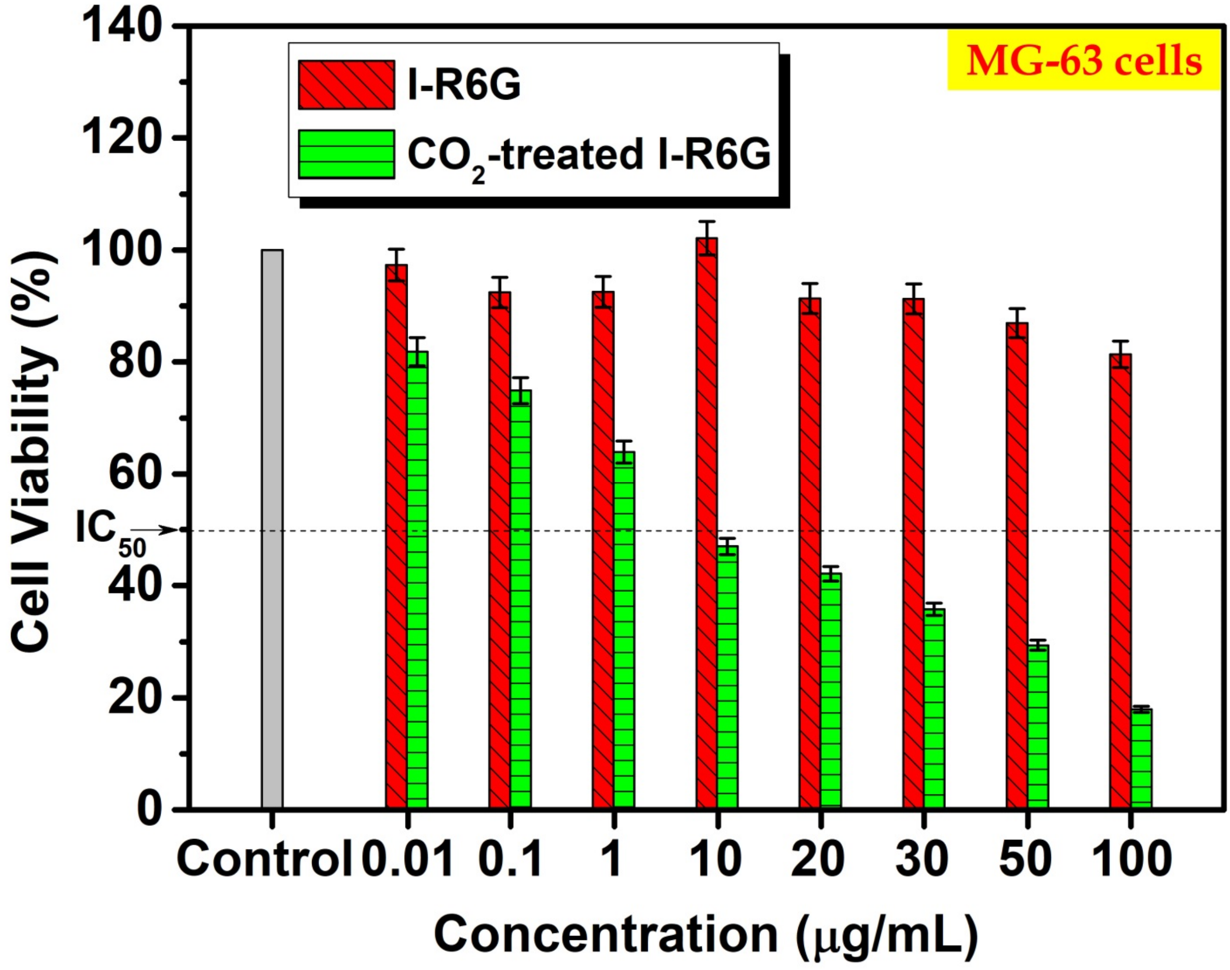

Figure S11: Cell viability of MG-63 cells *in vitro* after incubation with varying concentrations of pristine or CO_2-treated I-R6G (0.01−100 μg/mL) for 24 h.

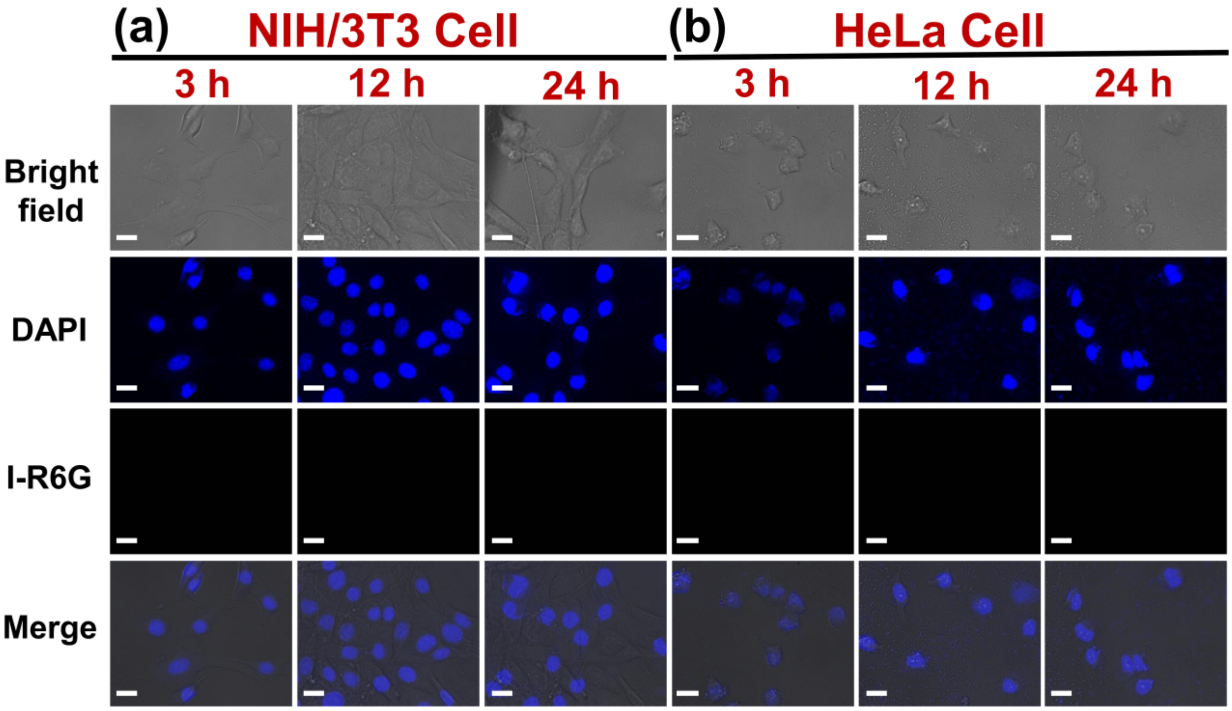

Figure S12: CLSM images of **(a)** NIH/3T3 and **(b)** HeLa cells cultured with pristine I-R6G at 37 °C for 3, 12 or 24 h. The scale bar in each image represents 20 μm.

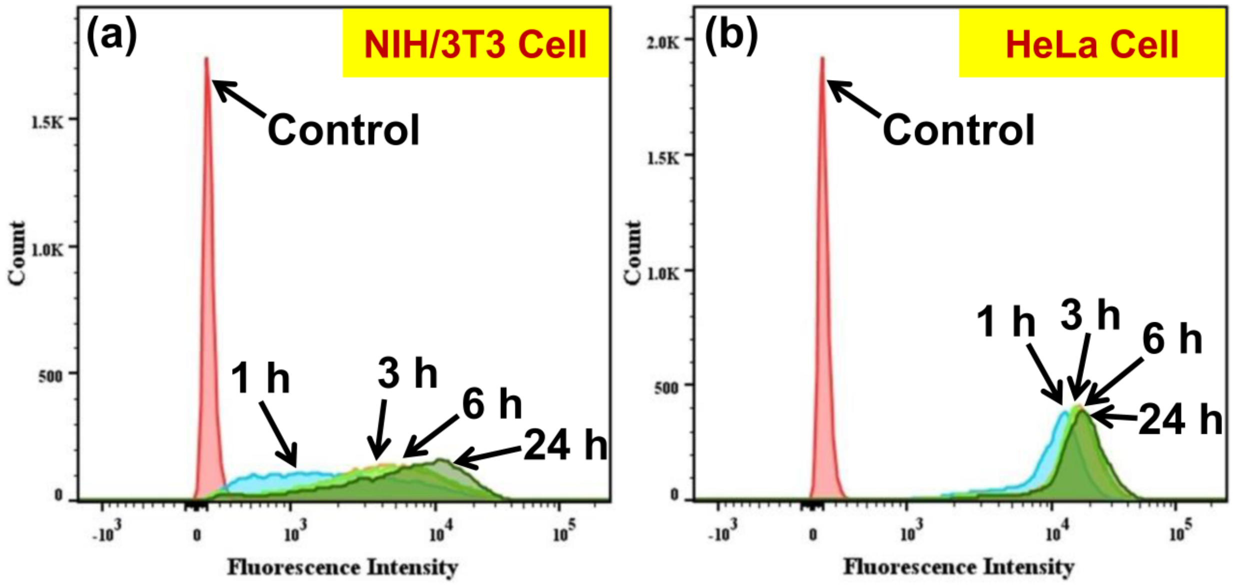

Figure S13: Flow cytometry histogram profiles of **(a)** NIH/3T3 and **(b)** HeLa cells cultured with R6G at 37 °C for 1, 3, 6 or 24 h.

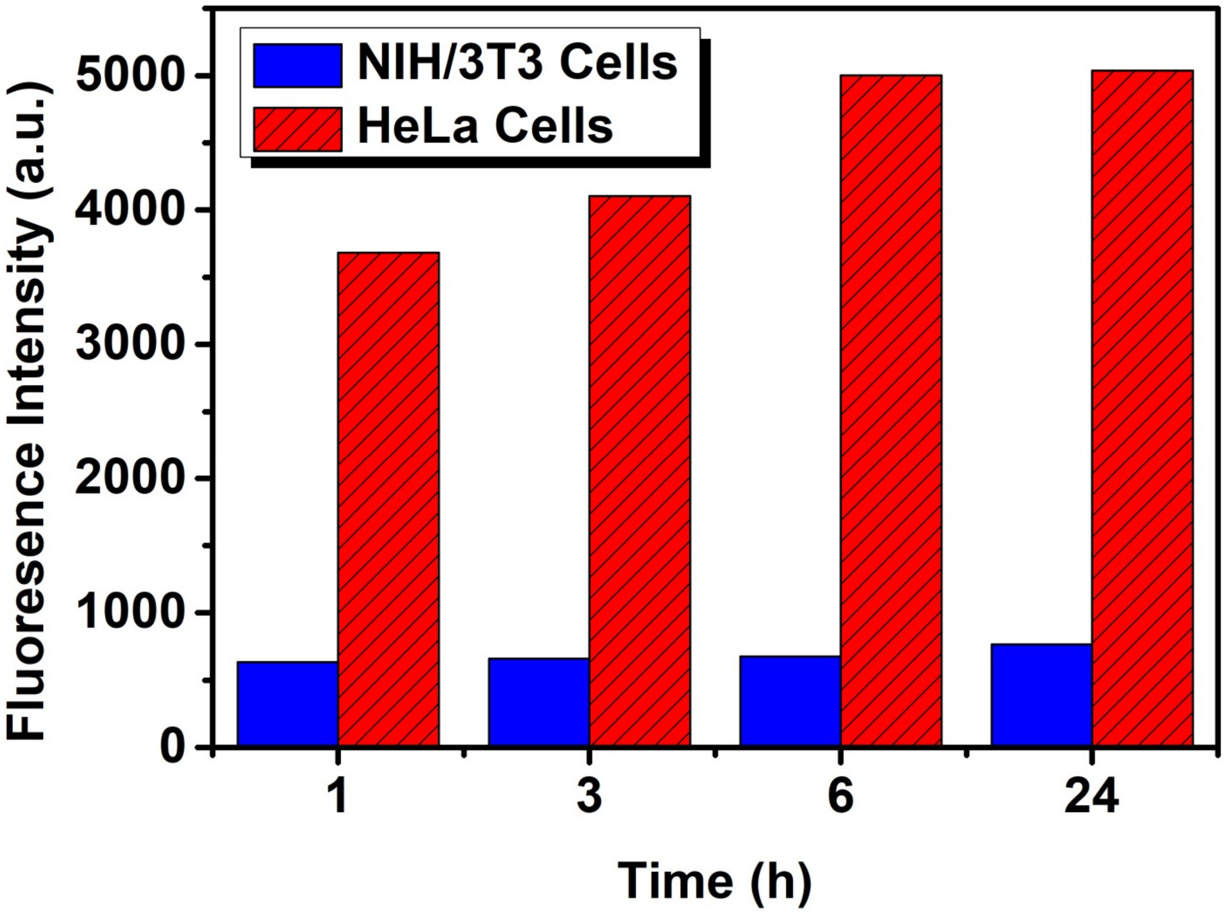

Figure S14: Flow cytometric analysis of the changes in the fluorescence intensities of NIH/3T3 and HeLa cells incubated with CO_2-treated I-R6G at 37 °C for 1, 3, 6 or 24 h.

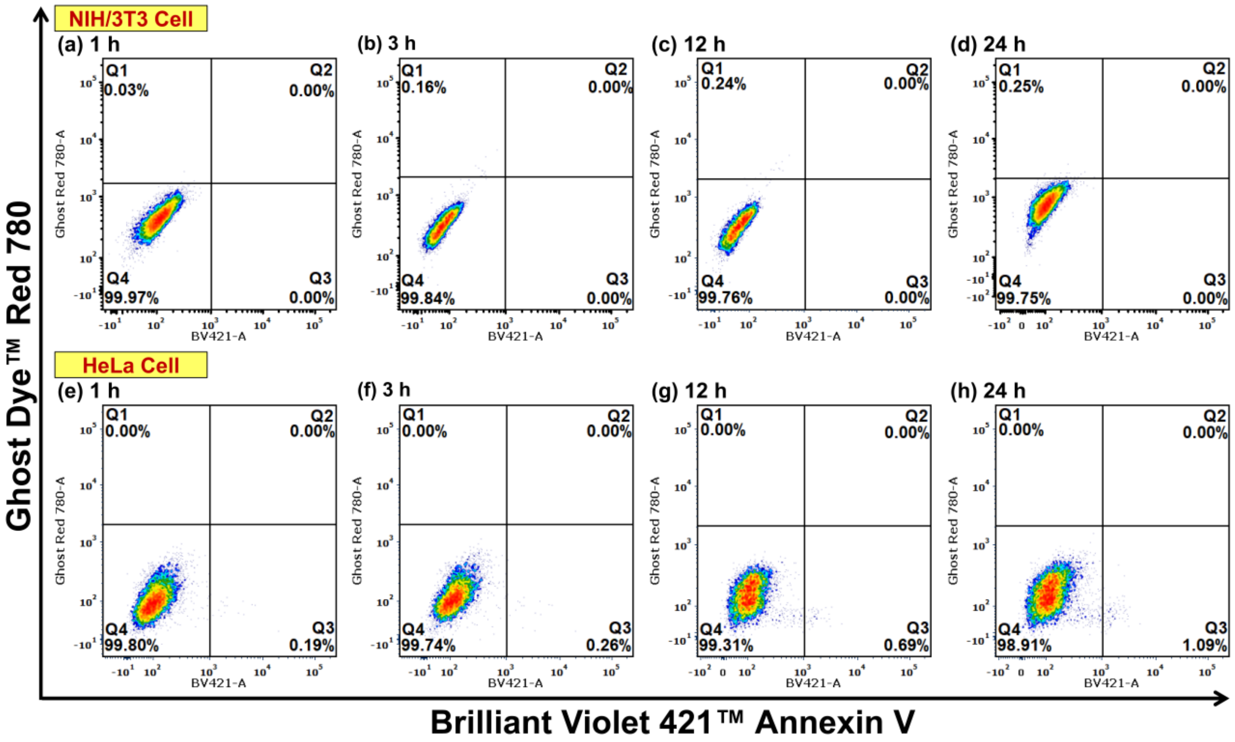

Figure S15: Representative dot plot diagrams generated by flow-cytometric analysis of **(a–d)** NIH/3T3 and **(e–h)** HeLa cells incubated with pristine I-R6G at 37 °C 1, 3, 12 or 24 h, then double stained with BV421 Annexin V and GDR-780.
